# Identification and characteristics of wheat *Lr* orthologs in three rye inbred lines

**DOI:** 10.1371/journal.pone.0288520

**Published:** 2023-07-13

**Authors:** Tomasz Krępski, Mateusz Olechowski, Izabela Samborska-Skutnik, Magdalena Święcicka, Agnieszka Grądzielewska, Monika Rakoczy-Trojanowska

**Affiliations:** 1 Department of Plant Genetics, Institute of Biology, Breeding and Biotechnology, Warsaw University of Life Sciences, Warsaw, Poland; 2 Educo BSH Sp. z o.o, Lublin, Poland; University of Delhi, INDIA

## Abstract

The genetic background of the immune response of rye to leaf rust (LR), although extensively studied, is still not well understood. The recent publication of the genome of rye line Lo7 and the development of efficient transcriptomic methods has aided the search for genes that confer resistance to this disease. In this study, we investigated the potential role of rye orthologs of wheat *Lr* genes (*Lr1*, *Lr10*, *Lr21*, *Lr22a*, and *RGA2/T10rga2-1A)* in the LR seedling-stage resistance of inbred rye lines D33, D39, and L318. Bioinformatics analysis uncovered numerous *Lr* orthologs in the Lo7 genome, namely, 14 *ScLr1*, 15 *ScRga2*, and 2 *ScLr21* paralogs, and 1 each of *ScLr10* and *ScLr22a* genes. The paralogs of *ScLr1*, *ScRga2*, and *ScLr21* were structurally different from one another and their wheat counterparts. According to an RNA sequencing analysis, only four wheat *Lr* gene orthologs identified in the Lo7 genome (*ScLr1_3*, *ScLr1_4*, *ScLr1_8*, and *ScRga2_6*) were differentially expressed; all four were downregulated after infection with compatible or incompatible isolates of *Puccinia recondita* f. sp. *secalis* (*Prs)*. Using a more precise tool, RT-qPCR, we found that two genes were upregulated at 20 h post-infection, namely, *ScLr1_4* and *ScLr1_8* in lines D33 and D39, respectively, both of which have been found to be resistant to LR under field conditions and after treatment with a semi-compatible *Prs* strain. We were unable to discern any universal pattern of gene expression after *Prs* infection; on the contrary, all detected relationships were plant genotype-, *Prs* isolate-, or time-specific. Nevertheless, at least some *Lr* orthologs in rye (namely, *ScLr1_3 ScLr1_4*, *ScLr1_8*, and *ScRga2_6*), even though mainly downregulated, may play an important role in the response of rye to LR.

## Introduction

Rye (*Secale cereale* L.), one of the most economically important cereals in Central and Eastern Europe, is strongly resistant to various biotic and abiotic stresses. Although regarded as disease tolerant, this crop can be attacked by up to 37 diseases, namely, 2 bacterial and 35 fungal ones (https://www.apsnet.org/edcenter/resources/commonnames/Pages/Rye.aspx). Among these diseases, leaf rust (LR; also termed brown rust), is caused by an airborne pathogen, the obligate biotrophic basidiomycete *Puccinia recondita* f. sp. *secalis* (*Prs*) [1,2]. Estimated yield losses due to leaf rust are typically up to 40% [3]; under conditions favorable for the development of infection, however, losses can be as high as 80% [4]. These losses are most often associated with decreases in the number of kernels per head and kernel weight, which are reduced by an average of 14% [3].

In rye, resistance to LR is usually controlled by a single, or occasionally two or three, dominant gene(s) [5]. These genes are referred to as *Pr* genes to distinguish them from their *Lr* counterparts, which confer resistance to LR in wheat. To date, 17 dominant *Pr* genes, including *Pr1-5*, *Pr-d–f*, *Pr-i–l*, *Pr-n*, *Pr-p*, *Pr-r*, and *Pr-t* [6,7], have been identified by Mendelian methods on both the long and short arms of five of the seven rye chromosomes (1R, 2R, 4R, 6R, and 7R). Most *Pr* genes have been mapped to chromosome 1R, which has been widely used as a chromatin source in wheat breeding [8]. This set complements the recently identified *Pr6* gene, located on chromosome 7RS [9].

Most of these genes, specifically, *Pr1–Pr5*, *Pr-d–f*, *Pr-n*, *Pr-p*, and *Pr-r*, protect rye against disease development [7] and may therefore be considered all-stage resistance genes [10]. Molecular studies focused on rye *Pr* genes are limited to two recently published reports by Vendelbo et al. [9,11]. Using a genome-wide association study, the authors mapped five LR resistance-associated quantitative trait loci on chromosome arms 1RS, 1RL, 2RL, 5RL, and 7RS, of which two located on chromosome arms 1RS and 7RS were of particular importance. The resistance-associated marker on chromosome arm 1RS was physically co-localized with molecular markers for *Pr3*. The region on chromosome arm 7RS contained a large number of nucleotide-binding leucine-rich repeat (NLR) genes, one of which, provisionally denoted *Pr6*, was similar (at the protein level) to the wheat LR resistance gene *Lr1* situated on chromosome arm 5DL. In contrast to these results, Milczarski et al. [12] identified chromosome 5R as the location of LR resistance-related quantitative trait loci with the highest impact on resistance to this disease.

The molecular aspects of rye response to LR have also been analyzed in relation to secondary metabolism. Święcicka et al. [13] recently found a relationship between expression patterns and levels of genes controlling benzoxazinoid (BX) biosynthesis and rye response to infection with LR. One of the examined genes, *ScBx4* encoding a cytochrome P450 monooxygenase, was induced in infected and mock-treated plants at all-time points (8, 17, 24, and 48 h post-treatment [hpt]). Because this observation was fully in line with an earlier finding that a single nucleotide polymorphism (ScBx4_1583) in the *ScBx4* gene is stably associated with the field resistance of adult plants to LR [14], the authors suggested that the identified polymorphism, ScBx4_1583, is associated with both non-race-specific adult-plant and seedling-stage resistance.

In general, the plant immune system includes two layers of pathogen–host interaction. The first layer generates pathogen-associated molecular pattern-triggered immunity (PTI) in host plants. To suppress PTI, pathogens secrete effectors into host plants. The R genes of host plants can recognize these effectors, thereby initiating the second layer: intracellular effector-triggered immunity (ETI) [15]. Plant resistance to disease (including LR) may be (1) pathogen race-specific, (2) non-race- and non-pathogen-specific, or (3) characterized by non-host resistance [16]. Consistent with the gene-for-gene model, the first class of resistance depends on single dominant R genes. The R genes encode proteins belonging to immune receptors of the nucleotide-binding leucine-rich repeat (NBS-LRR) class, which recognize Avr proteins produced by pathogens [17,18]. Most R genes have three or four exons. Exons one and two respectively encode the toll/interleukin-1 receptor (TIR)/coiled-coil (CC) domain (TIR mainly present in dicots, and CC in both di- and monocots) and the NBS domain (also called the NB-ARC domain, for nucleotide-binding adaptor shared by APAF-1, R proteins, and CED-4) on the C terminal, whereas the third (and fourth) exon(s) encode the leucine-rich repeat (LRR) domain on the N terminal [19]. Exceptions exist, however; for example, Andersen et al. [20] discovered five NB-ARC-encoding genes with a TIR domain but no LRR domain in wheat. In cereals, the NBS domain is composed of six conserved motifs: P-loop, RNBS-A, Kinase-2, Kinase-3a, RNBS-C, and GLPL [21].

Plant R genes activate a defense response and are differentially expressed (usually upregulated) after pathogen infection, regardless of the plant–pathogen interaction [22]. Nevertheless, R-gene downregulation has occasionally been observed, such as in *Arabidopsis* after infection with two pathogens (*Hyaloperonospora parasitica* and *Erisyphe cichoracearum*) [23], and in hops and tobacco in compatible reactions with *Verticillium albo-atrum* [24] and *Ralstonia solanacearum* [25], respectively. An analysis performed by Peng and Yang [26] revealed that R genes may not be induced at all, especially in the incompatible reaction between wheat and *Puccinia triticina*.

Unlike the scenario in rye, several R genes in wheat (the closest relative of rye) that confer resistance to LR at the seedling stage have been sequenced and molecularly characterized. These genes are as follows: *Lr1* on chromosome 5DL [27], *Lr10* on chromosome 1AS (together with an additional gene essential to its function, *RGA2/T10rga2-1A* [28,29]), *Lr13/LrLC10* on chromosome 2BS [30], *Lr21* on chromosome 1DS [31,32], and *Lr22a* on chromosome 2DS [33]. Investigation of their differential expression is limited to several works but none employed RNA-sequencing (RNA-seq) or quantitative reverse transcription PCR (RT-qPCR) methods. As shown by Cloutier et al. [27], the expression level of *Lr1*, as determined by reverse transcription PCR (RT-PCR) analysis, corresponded with the phenotype (resistant or susceptible). Kumar et al. [34], using the Affymetrix GeneChip® Wheat Genome Array, demonstrated that *Lr1* triggers early initiation of a set of signaling cascades that leads to activation of defense- and stress response-related genes. Information on the expression of the *T10rga1/Lr10* gene has been reported by Feuillet et al. [28], who observed that overexpression of *Lr10*, as measured by northern blotting, was correlated with enhanced resistance to LR and complete suppression of rust sporulation. No expressional data are available for three other aforementioned genes.

The aim of this study was to identify and characterize orthologs of wheat genes with a confirmed role in the immune response to LR in the newly released rye Lo7 genome [35] and in three rye inbred lines (D39, D33, and L318). We also conducted structural and expression analyses to determine if the orthologous genes are affected by LR at the seedling stage.

## Materials and methods

### Plant materials

We used three unrelated rye inbred lines as plant materials in experiments: L318, bred by the Department of Plant Genetics, Breeding and Biotechnology, Warsaw University of Life Sciences (Warsaw, Poland), and D33 and D39, both developed by Danko Plant Breeding, Ltd. (Poland). The inbred lines were selected according to the results of experiments described in two published studies, namely, measurements of BX contents of field-grown plants after a natural vernalization period [14] (S1 Table) and comparisons of seedling-stage responses to artificial infection with a partially compatible strain of *Prs* [13].

### Pathogen selection

In experiments in this study, rye inbred lines were infected with four *Prs* isolates selected by preliminary screening of 15 single-spore isolates. The selections were based on detached-leaf inoculations (performed as described by Święcicka et al. 2020 [13]) followed by confirmation *in planta*. Each isolate was re-tested on twenty-four 20-day-old seedlings (3 pots × 8 plants) of each inbred line cultivated under a 16-h/8-h photoperiod, 50% humidity, and 22°C (day) and 18°C (night) temperatures. Immediately after infection, plants were transferred to a room maintained at 18°C, covered with black boxes to preserve darkness and 100% humidity, and incubated for 24 h. The specificity of the plant–pathogen interaction, that is, whether the interaction between the plant and the pathogen was compatible (medium to large uredinia, with chlorosis; or medium to large uredinia, with little or no chlorosis; infection types 3 and 4 according to the Murphy scale [36] (S1 Fig) or non-compatible (no visible signs of infection, chlorosis, and necrotic flecking; infection types 0 and 0; according to the Murphy scale [36] (S1 Fig) was evaluated 10 days after inoculation.

Finally, four isolates derived from single spores were selected for the main experiment. Two isolates, no. 1/1.6 (non-compatible) and no. 83/2/2.2_5x (compatible), were used for inoculating lines D33 and D39. Two other isolates, no. 81/r/5_5x (non-compatible) and no. 88/o/1_5x (compatible), were applied to line L318. The infection scheme and plant–pathogen interactions are presented in Table 1.

**Table 1:** Types of interactions between rye inbred lines and *Prs* isolates.

*Prs* isolate Rye line	No. 1/1.6	No. 83/2/2.2_5x	No. 81/r/5_5x	No. 88/o/1_5x
D33	non-compatible	compatible		
D39	non-compatible	compatible		
L318			non-compatible	compatible

### Experimental design

Seeds of the three rye inbred lines (D33, D39, and L318) were initially sown in Petri dishes lined with wet tissue paper and left in the dark for 2 days at 22°C. Germinating seeds were then transferred into 12-cm diameter plastic pots (10 seedlings per pot) filled with sterilized peat substrate and maintained for 10 days in a growth chamber at 22°C under a 16-h light/8-h dark photoperiod at an illumination intensity of 60 μmol m^−2^ s^−1^.

Experiments were performed with three biological replicates, each comprising one pot with 10 plants, and two time points (20 hours post treatment, hpt and 36 hpt). The time points were selected based on our previous research [13] which showed that the highest number of spores forming haustorium mother cells (HMC) are observed between 20th and 36th hpt. Each rye inbred line was represented by 18 pots: 6 with mock-treated controls, 6 inoculated with a compatible *Prs* isolate, and 6 treated with a non-compatible isolate.

### *Prs*- and mock treatments

Spores from a single-spore isolate of *Prs* were suspended in Novec-7100 engineered fluid (at a density of 1 mg cm^−3^). Plant infection was carried out using brown glass diffusers (Roth, Basel, Switzerland). Control (mock) plants were treated in the same way as plants inoculated with *Prs*, except that the applied Novec-7100 engineered fluid lacked *Prs* spores. After treatment, plants were maintained as described above.

### Sampling of plant materials

Plant material was collected two times: 20 hpt and 36 hpt. After sampling, plant tissue was immediately frozen in liquid nitrogen and stored in a freezer at −80°C.

### DNA extraction for *ScLr* gene identification and sequencing

To identify wheat *Lr* orthologs in rye (hereafter termed *ScLr* genes), DNA was isolated from young leaves of the three rye lines (D33, D39, and L318) using a Plant and Fungi DNA Purification kit (EurX, Gdańsk, Poland). DNA quantity and quality were checked on a NanoDrop 2000 spectrophotometer and by visualization on 1% agarose gels.

### RNA extraction for expression analysis

Total RNA was extracted from approximately 100 mg of frozen leaves of mock- and *Prs*-treated rye lines (D33, D39, and L318) using a mirVana miRNA Isolation kit plus Plant RNA Isolation Aid (Thermo Fisher Scientific, Waltham, MA, USA). The isolated RNA was dissolved in 50 μl RNase-free water, and RNA yield and purity were estimated using a NanoDrop 2000 spectrophotometer and a Qubit 4 fluorometer. Subsequently, the extracted RNA was treated with DNase I (Thermo Fisher Scientific, Waltham, MA, USA) for removal of genomic DNA contamination in accordance with the manufacturer’s protocol and stored at −80°C.

### Identification and characterization of rye *Lr* orthologs by bioinformatics analysis

To identify rye *Lr* orthologs, coding sequences (CDSs) of known wheat *Lr* genes, including *Lr1*, *Lr10*, *RGA2/T10rga2-1A*, *Lr21*, and *Lr22a*, were first aligned with the genome of rye line Lo7 [35] using the standalone NCBI BLAST+ v2.9.0 program. The best matches (dc-megablast: *e*-value ≤ 1 × 10^−6^, sequence identity ≥ 70%, and query coverage per HSP ≥ 30%) were chosen for further analysis. The identified rye *Lr* orthologs were named by prefixing *Sc* to the *Lr* gene name used as a query in the BLAST search and appending numbers that successively increased as the bit score parameter for the BLAST results decreased. In other words, the number 1 always corresponded to the most similar ortholog to the wheat *Lr* gene, whereas the highest number was assigned to the ortholog with the lowest similarity. The structures of the identified wheat *Lr* gene orthologs in rye (*ScLr* genes) were collected from the gff3 file (the version containing all predicted genes) available at the same site as the above-mentioned rye Lo7 genome. In addition, conserved domains of each analyzed *ScLr* gene were identified using the NCBI Conserved Domains Database [37] and visualized together with exon–intron structures using GSDS v2.0 [38]. A phylogenetic tree of ScLr proteins was reconstructed by maximum likelihood using the Phylogeny.fr platform [39]. ScLr protein transmembrane domains and subcellular locations were predicted using the TMHMM v2.0 server (http://www.cbs.dtu.dk/services/TMHMM/) and TargetP v2.0 (Organism: Plant) [40], respectively.

### RNA-sequencing analysis

Total RNAs extracted as described above from rye plants were sent to Genomed S.A. (Warsaw, Poland) for RNA-seq, assembly, and primary gene expression analysis. In brief, RNA-seq libraries were prepared using a NEBNext Ultra II Directional RNA Library Prep Kit for Illumina (NEB, USA). In total, 54 cDNA libraries were sequenced (S2 and S8B Tables) in PE150 mode on a NovaSeq 6000 system (Illumina). The resulting sequencing reads were filtered in Cutadapt v3.0 (with the parameters: minimum-length = 15 and quality-cutoff = 25) [41], and quality reports were generated using FASTQC v0.11.8 [42]. After trimming with Cutadapt, the average transcriptome coverage by RNA-seq was 110× per library with an average read length of 136 bp. All reads ≥ 20 bp were then mapped to the *S*. *cereale* Lo7 reference genome [35] using Hisat2 v2.2.0 [43] with the library preparation option ‘rna-strandness RF’. Next, paired reads mapped to individual genes were counted in HTseq [44] using the ‘—stranded = reverse’ option to differentiate transcript strands. For all other HTseq settings the default option was used. Gene annotations were assigned according to the gene description file (gff3 file, the version containing all predicted genes) from the Lo7 *S*. *cereale* genome [35], and final results were compiled in R v3.6.3 [45] (R Core Team 2020) using DESeq2 v1.26.0 [46]. Finally, significant differentially expressed genes (DEGs) were identified, grouped, and analyzed using Microsoft Excel v2016 (Microsoft, USA).

The following criteria were used for identification of significant DEGs: |log2 fold change| ± 0.5 and false discovery rate < 0.05. No cut-off parameter for the average value of the raw read counts was used, because some NBS domain-containing genes are known to be extremely weakly expressed [26] and thus it was not intended to exclude these genes from the analysis. RNAseq (fastq) data has been deposited in NCBI (http://www.ncbi.nlm.nih.gov/bioproject/888031; BioProject 208 ID PRJNA888031).

### Primer design and gene sequencing

Primers for amplification, sequencing, and RT-qPCR analysis of the studied genes (*ScLr1_3*, *ScLr1_4*, *ScLr1_8*, and *ScRga2_6*) in the three rye lines (D33, D39, and L318) were designed using NCBI Primer-BLAST [47]. The rye Lo7 genome assembly (GenBank: GCA_902687465.1) was used as a target template for designing primers and checking primer-pair specificity. To assess designed primer quality, we used PCR Primer Stats [48] to test for the presence of hairpins and OligoAnalyzer Tool (Integrated DNA Technologies, USA) to check for potential homo- and heterodimer formation. The complete list of primers used in this study is provided in S4 Table. The PCR amplicons (ranging from 837 to 1087 bp) obtained from rye lines D33, D39, and L318 were sent to Genomed S.A. for DNA sequencing by the Sanger method. The generated sequences were analyzed and manually annotated in SnapGene Viewer 5.2.4 (GSL Biotech, USA), and sequence comparisons and DNA fragment assembly were carried out in Clustal Omega [49]. Primers for RT-qPCR were then designed to amplify the sequenced gene regions.

### RT-qPCR analysis of selected orthologs of wheat *Lr* genes

The RNA-seq-based expressions of four selected *Lr* gene orthologs in rye (*ScLr1_3*, *ScLr1_4*, *ScLr1_8*, and *ScRga2_6*) were validated by RT-qPCR. For the RT-qPCR analysis, 1 μg of total RNA (extracted as described in section RNA extraxction for expression analysis) was used as the template for cDNA synthesis with a RevertAid First Strand cDNA Synthesis kit (Roche, Basel, Switzerland). The analysis was performed in 96-well plates, with three biological and two technical replicates, on a LightCycler 96 Real Time System (Roche). The RT-qPCR cycling conditions were as follows: 10 min of denaturation at 95°C followed by 35 amplification cycles (10 s at 95°C, 10 s at 57°C, and 15 s at 72°C). The amplifications were performed in 20-μl reaction volumes consisting of 4 μl cDNA (5 ng/μl), 1 μl of each gene-specific primer (5 mM), 4 μl RNase-free water, and 10 μl FastStart Essential DNA Green Master Mix (Roche). Relative expression levels were calculated according to the 2^−ΔΔCt^ method [50] using the expressions of the barley actin gene *HvAct* (GenBank: AY145451 [13]) and the ADP-ribosylation factor (arf) gene *TaADP-RF* (GenBank: BJ242826.1; primer pairs designed by Giménez et al. [51]) as references. Significant differences in gene expression between *Prs*-treated rye plants and controls (untreated plants) were identified using the REST tool [52].

A graphical presentation of all experiments is shown on S3 Fig.

## Results

### Identification and characterization of *Lr* orthologs in the Lo7 genome

Orthologs of the following wheat R genes conferring seedling-stage resistance to LR were identified in the rye Lo7 genome: *Lr1*, *Lr10*, RGA2/*T10rga2-1A*, *Lr21*, and *Lr22a*. Our bioinformatics analysis uncovered several orthologs of *Lr1* (*ScLr1_1*–*ScLr1_14*) and *Rga2* genes (*ScRga2_1*–*ScRga2_15*), two orthologs of *Lr21* (*ScLr21_1* and *ScLr21_2*), and one ortholog each of *Lr10* (*ScLr10*) and *Lr22a* (*ScLr22a*) (Table 2).

**Table 2:** Identification of wheat *Lr* gene orthologs (*ScLr* genes) in rye Lo7 genome. Rye orthologs DNA sequence identity ≥ 70% and e-value ≤ 10^−6^, query coverage per HSP ≥ 30%. Abbreviation “qcov” refers to query coverage per HSP (High Scoring Pair).

Known *Lr* gene	Rye ortholog ID	Rye cortholog name	Rye chromosome	% identity/qcov	bit score	E-value*
*Lr1* (GenBank: EF439840.1)	SECCEUnv1G0527350	*ScLr1_1*	Un	87/96	4745	0
	SECCEUnv1G0527300	*ScLr1_2*	Un	87/89	4414	0
	SECCE7Rv1G0454480	*ScLr1_3*	7R	89/63	3368	0
	SECCE7Rv1G0454510	*ScLr1_4*	7R	89/63	3362	0
	SECCE7Rv1G0454380	*ScLr1_5*	7R	89/63	3296	0
	SECCE7Rv1G0454390	*ScLr1_6*	7R	88/63	3288	0
	SECCE7Rv1G0454490	*ScLr1_7*	7R	88/63	3269	0
	SECCEUnv1G0527330	*ScLr1_8*	Un	90/48	2691	0
	SECCE7Rv1G0454400	*ScLr1_9*	7R	89/49	2573	0
	SECCEUnv1G0527290	*ScLr1_10*	Un	86/49	2355	0
	SECCE1Rv1G0053510	*ScLr1_11*	1R	74/74	2003	0
	SECCE6Rv1G0447940	*ScLr1_12*	6R	71/52	1052	0
	SECCE6Rv1G0447930	*ScLr1_13*	6R	71/52	1048	0
	SECCEUnv1G0561580	*ScLr1_14*	Un	72/45	995	0
*Lr10* (GenBank: AY270157.1)	SECCE6Rv1G0440520	*ScLr10*	6R	70/40	445	1.8E-123
RGA2/*T10rga2-1A* (GenBank: AY270159.1)	SECCE1Rv1G0000300	*ScRga2_1*	1R	75/73	1785	0
	SECCE7Rv1G0483290	*ScRga2_2*	7R	74/74	1593	0
	SECCE6Rv1G0437290	*ScRga2_3*	6R	73/74	1480	0
	SECCE3Rv1G0156830	*ScRga2_4*	3R	71/87	1472	0
	SECCE6Rv1G0437180	*ScRga2_5*	6R	72/74	1431	0
	SECCE6Rv1G0437270	*ScRga2_6*	6R	72/74	1360	0
	SECCE3Rv1G0156840	*ScRga2_7*	3R	71/74	1241	0
	SECCE4Rv1G0265930	*ScRga2_8*	4R	77/31	834	0
	SECCE4Rv1G0265620	*ScRga2_9*	4R	76/33	822	0
	SECCE4Rv1G0265660	*ScRga2_10*	4R	75/33	772	0
	SECCE5Rv1G0307590	*ScRga2_11*	5R	72/32	681	0
	SECCE1Rv1G0055240	*ScRga2_12*	1R	72/30	569	8.1E-161
	SECCE5Rv1G0306370	*ScRga2_13*	5R	71/30	540	3.9E-152
	SECCE5Rv1G0307570	*ScRga2_14*	5R	71/30	534	5.8E-150
	SECCE6Rv1G0437260	*ScRga2_15*	6R	70/36	329	1.28E-88
*Lr21* (GenBank: FJ876280.1)	SECCE1Rv1G0002910	*ScLr21_1*	1R	82/82	2675	0
	SECCE1Rv1G0002930	*ScLr21_2*	1R	75/62	1361	0
*Lr22a* (GenBank: KY064064.1)	SECCE4Rv1G0286050	*ScLr22a*	4R	73/90	1410	0

Un–unknown chromosome.

*^)^NCBI BLAST/BLAST+ rounded any e-values smaller than 1.0E-180 to 0.

**Fig 1:**
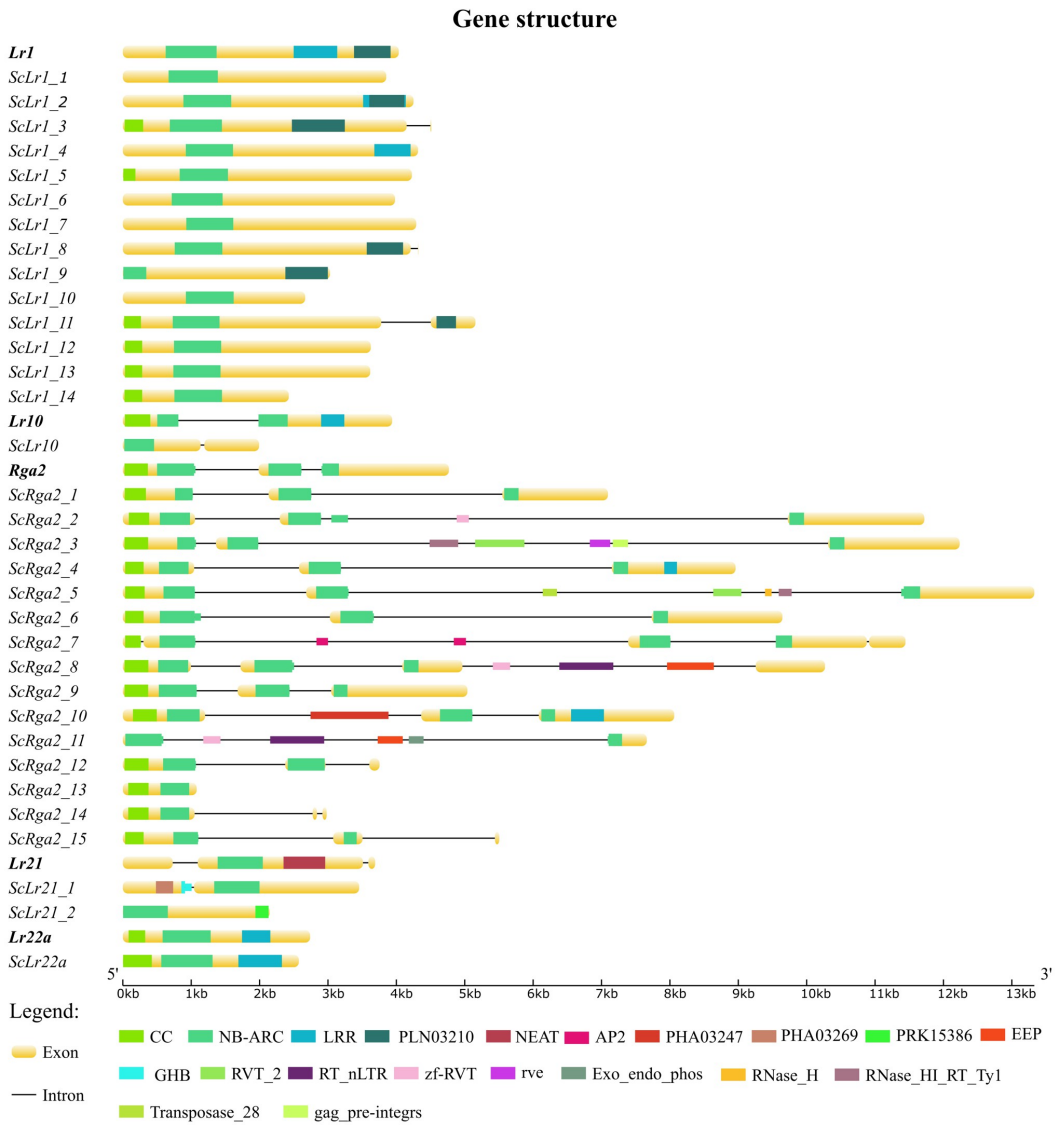
Structure of identified wheat *Lr* gene orthologs in rye (*ScLr* genes). Conserved domains localization for each gene was marked: CC–Coiled-coil domain, NB-ARC–NB-ARC domain, PLN03210 –PLN03210 domain (Resistant to P. syringae 6; Provisional), LRR–Leucine-rich repeat domain, NEAT–NEAr Transport domain, AP2—AP2 domain, PHA03269—PHA03269 domain, PHA03269—PHA03269 domain, EEP—Exonuclease-Endonuclease-Phosphatase domain, GHB—Glycoprotein hormone beta chain homologues domain, RVT_2—Reverse transcriptase domain, RT_nLTR—Non-LTR (long terminal repeat) retrotransposon and non-LTR retrovirus reverse transcriptase (RT) domain, zf-RVT—zinc-binding in reverse transcriptase, rve—Integrase core domain, Exo_endo_phos—Endonuclease/Exonuclease/phosphatase family domain, RNase_H—Ribonuclease H domain, RNase_HI_RT_Ty1—Ty1/Copia family of RNase HI in long-term repeat retroelements domain, Transposase_28—transposase 28 domain, gag_pre-integrs—GAG-pre-integrase domain.

Most *ScLr1* genes were found to have one exon in the CDS region, similar to wheat *Lr1*. The exceptions were *ScLr1_3*, *ScLr1_8*, and *ScLr1_11*, with an intron near the end of the CDS (Fig 1). Wheat *Lr1* genes also have an intron; however, this intron in wheat is located after the stop codon, at the beginning of the 3′ UTR [27]. Although most *ScLr1* genes had CDSs of similar size to those of *Lr1* genes, the CDSs of three (*ScLr1_9*, *ScLr1_10*, and *ScLr1_14*) were definitely shorter than in their wheat orthologs (S5 Table). Bioinformatic analysis indicated that several rye *ScLr1* paralogs (*ScLr1_1*, *ScLr1_2*, *ScLr1_4*, *ScLr1_6*, and *ScLr1_7*) encode a signal peptide in their N-terminal, similar to *Lr1* gene (S7 Table). The identified amino acid sequences forming the signal peptide in proteins encoded by the *Lr1* gene and *ScLr* genes are highly conserved (S4 Fig). No signal peptide was detected in the remaining *ScLr1* genes. In addition, *in silico* analysis revealed that the protein encoded by the *ScLr1_7* gene is a transmembrane protein (S6 Table).

We found that the rye *ScLr10* gene was structurally different from its wheat ortholog: it had a very small intron (55 bp long), whereas the *Lr10* intron had a total length of 1,172 bp (S5 Table). Moreover, the *Lr10* gene sequence was approximately twice the size of the *ScLr10* gene. *ScLr10* also lacked CC and LRR domains and only possessed the NB-ARC domain (Fig 1). Despite these differences, the CDSs of the two genes shared approximately 70% DNA sequence similarity.

Similar to wheat *Rga2* genes, all but four rye *ScRga2* gene paralogs had two introns. In particular, *ScRga2_7*, *ScRga2_8*, *ScRga2_11*, and *ScRga2_13* had four, three, one, and no introns, respectively. *ScRga2* genes varied significantly in length but could be divided into two groups on the basis of CDS length: 1) *ScRga2_1*–*ScRga2_10*, with CDSs ranging from 3,186 to 3,927 bp, similar to *Rga2* (3,510 bp), and 2) *ScRga2_11*–*ScRga2_15*, with much smaller CDSs ranging from 1,125 to 1,590 bp. The length of the entire *Rga2* gene region varied greatly among paralogs, from 1,080 to 13,329 bp, mainly because of the presence or absence of large introns typically harboring protein domains related to transposable elements. Unlike other identified *ScLr* genes, which have one NB-ARC domain, all *ScRga2* paralogs except for *ScRga2_11*, *ScRga2_13*, *ScRga2_14*, and *ScRga2_15* had two (the first one fragmented) (Fig 1). In addition, all rye *ScRga2* genes except for *ScRga2_11* had a CC domain like wheat *Rga2* genes.

The first paralog of *ScLr21* (*ScLr21_1*) was structurally much more similar to *Lr21* in comparison with the second one (*ScLr21_2)*. *ScLr21_1* had a size similar to that of *Lr21* and, like the *Lr21* gene, was predicted to encode a transmembrane peptide (S2 Fig). *Lr21* has two introns, whereas *ScLr21* had only one. *ScLr21_2* lacked introns and was approximately half the length of *Lr21*. Both *ScLr21* paralogs and *Lr21* had one NB-ARC domain; in *ScLr21_2*, however, this domain was located at the beginning of the gene.

*Lr22a* and its homolog, *ScLr22a*, were found to be canonical CNL genes, both having CC, NB-ARC, and LRR domains. These two genes had similar sizes and possessed only one exon, but their CDS similarity was not very high (approximately 73%).

A phylogenetic analysis revealed that the paralogs of each ScLr protein were more closely related to one another than to other ScLr proteins, thus suggesting their status as independent gene families (S5 Fig). The same situation was observed for Lr proteins, which were more similar to their rye orthologs than to other wheat Lr proteins. According to our phylogenetic tree, however, the Rga2 clade was most similar to the Lr10 clade, whereas the ScLr21 clade was most closely related to the ScLr1 protein family. The results of the phylogenetic analysis were compatible with our BLAST-based DNA sequence comparison (Table 2) except that the protein encoded by the Rga2 gene was more to similar to ScRga2_2 and ScRga2_11 proteins than to the ScRga2_1 protein according to the phylogenetic analysis.

### RNA-seq-based gene expressions

Among the orthologs of wheat *Lr* genes identified in the Lo7 genome, four genes (*ScLr1_3*, *ScLr1_4*, *ScLr1_8*, and *ScRga2_6*) were differentially expressed. In total, DEGs were detected in six different comparisons, most often at 36 hpt (Table 3). Expression levels of all four genes decreased after infection with *Prs*, both with compatible (D33 C vs. MT at 20 hpt and L318 C vs. MT at 36 hpt) and non-compatible (L318 NC vs. MT at 36 hpt and D33 NC vs. MT at 20 hpt) isolates. In both cases, expression level reductions were more pronounced following infection with the compatible isolate than the non-compatible one. The expression of any of the other rye orthologs of the *Lr* genes (including *Lr10*, *Lr21* and *Lr22a*) was not significantly changed.

**Table 3:** Differentially expressed *Lr* gene orthologs in rye lines D33, D39, and L318 after treatment with different *Prs* isolates (compatible [C] or non-compatible [NC]) or a mock treatment (MT). Full RNA-seq results for Lr genes orthologs in all three rye lines are provided in the S8A Table.

*Prs* treatment comparison	Gene ID	Gene name	log2 Fold Change	FDR adjusted p-value
**L318 NC** **vs** **MT 36 hpt**	SECCE7Rv1G0454480	*ScLr1_3 (chr7R6)*	-0.73	0.0253
**D39 C** **vs** **NC 36 hpt**	SECCE7Rv1G0454510	*ScLr1_4 (chr7R7)*	-2.83	0.0425
**D33 NC** **vs** **MT 20 hpt**	SECCEUnv1G0527330	*ScLr1_8 (chrUn3)*	-0.83	0.0236
**D33 C** **vs** **MT 20 hpt**	SECCEUnv1G0527330	*ScLr1_8 (chrUn3)*	-1.06	0.0344
**L318 C** **vs** **MT 36 hpt**	SECCE6Rv1G0437270	*ScRga2_6*	-0.78	0.0143
**L318 C** **vs** **NC 36 hpt**	SECCE6Rv1G0437270	*ScRga2_6*	-0.74	0.0132

Each treatment included three biological replicates. Gene expression was analyzed in two time points (20 hpt and 36 hpt). For identification of significant DEGs, the following criteria were used: |log2 fold change|  ± 0.5 and false discovery rate < 0.05.

### Identification of selected differentially expressed *ScLr1* and *ScRga2* variants in genomes of rye inbred lines L318, D33, and D39

Identification of the genes that were differentially expressed in response to LR infection, as determined by RNA-seq, in the genomes of the inbred lines D33, D39, and L318 by PCR amplification was conducted (Table 3). *ScLr1_3*, which was differentially expressed in line L318 according to RNA-seq, could only be amplified from gDNA in this same line, whereas *ScLr1_4* was amplified in all tested lines. The variant *ScLr1_8*, identified by RNA-seq in line D33, could not be amplified from the gDNA template of this line but was detected in the other two lines. The reasons for this result will be discussed later. The final gene, *ScRga2_6*, was identified both by RNA-seq and gDNA sequencing, but only in line L318 (Table 4).

**Table 4:** Identification of genes selected on the basis of RNA-seq analysis in the genomes of three rye inbred lines.

Gene name	Amplification on gDNA template		
	D33	D39	L318
*ScLr1_3*	-	-	+
*ScLr1_4*	+	+	+
*ScLr1_8*	-	+	+
*ScRga2_6*	-	-	+

„+” amplicon, „-” no amplicon.

### RT-qPCR validation of *in vivo* expressions of DEGs identified by RNA-seq analysis

The expressions of genes differentially expressed in all 18 *Prs* treatment comparisons according to RNA-seq (S3 Table) were validated by RT-qPCR. Gene variants that were absent from the genomes of particular rye lines were excluded.

**Fig 2:**
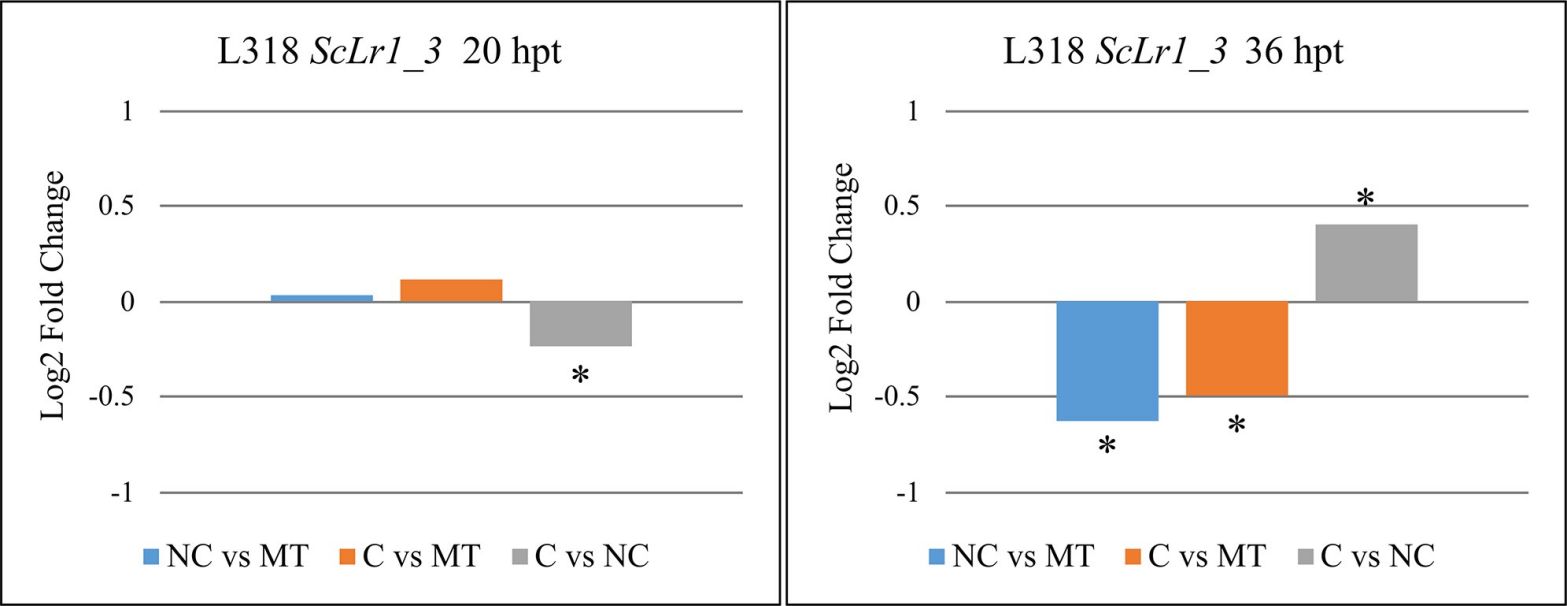
Log2 fold changes in the expression of the *ScLr1_3* gene in rye line L318 after treatment with different *Prs* isolates (compatible [C] or non-compatible [NC] strains) relative to the other treatment and a mock-treated control. The *ScLr1_3* gene was absent from rye lines D33 and D39. Plant material for the expression analysis was collected twice: At 20 and 36 hpt. Asterisks indicate significant differences in expression within a given comparison (*p* < 0.05).

The RT-qPCR analysis confirmed that *ScLr1_3* expression was significantly downregulated in line L318 at 36 hpt after treatment with a non-compatible *Prs* strain (Fig 2). Unlike the RNA-seq results, however, the RT-qPCR analysis revealed that this gene was also downregulated at this same time point in L318 after treatment with a compatible strain. No significant differences in *ScLr1_3* expression were observed between the mock treatment and *Prs* treatments, regardless of strain type, at 20 hpt, which was consistent with the RNA-seq data. With respect to the two strain types, *ScLr1_3* gene expression was significantly higher at 20 hpt and lower at 36 hpt in line L318 infected with a non-compatible strain compared with a compatible strain. Because *ScLr1_3* was not present in D33 and D39, RT-qPCR verification of this gene was not performed in those lines.

**Fig 3:**
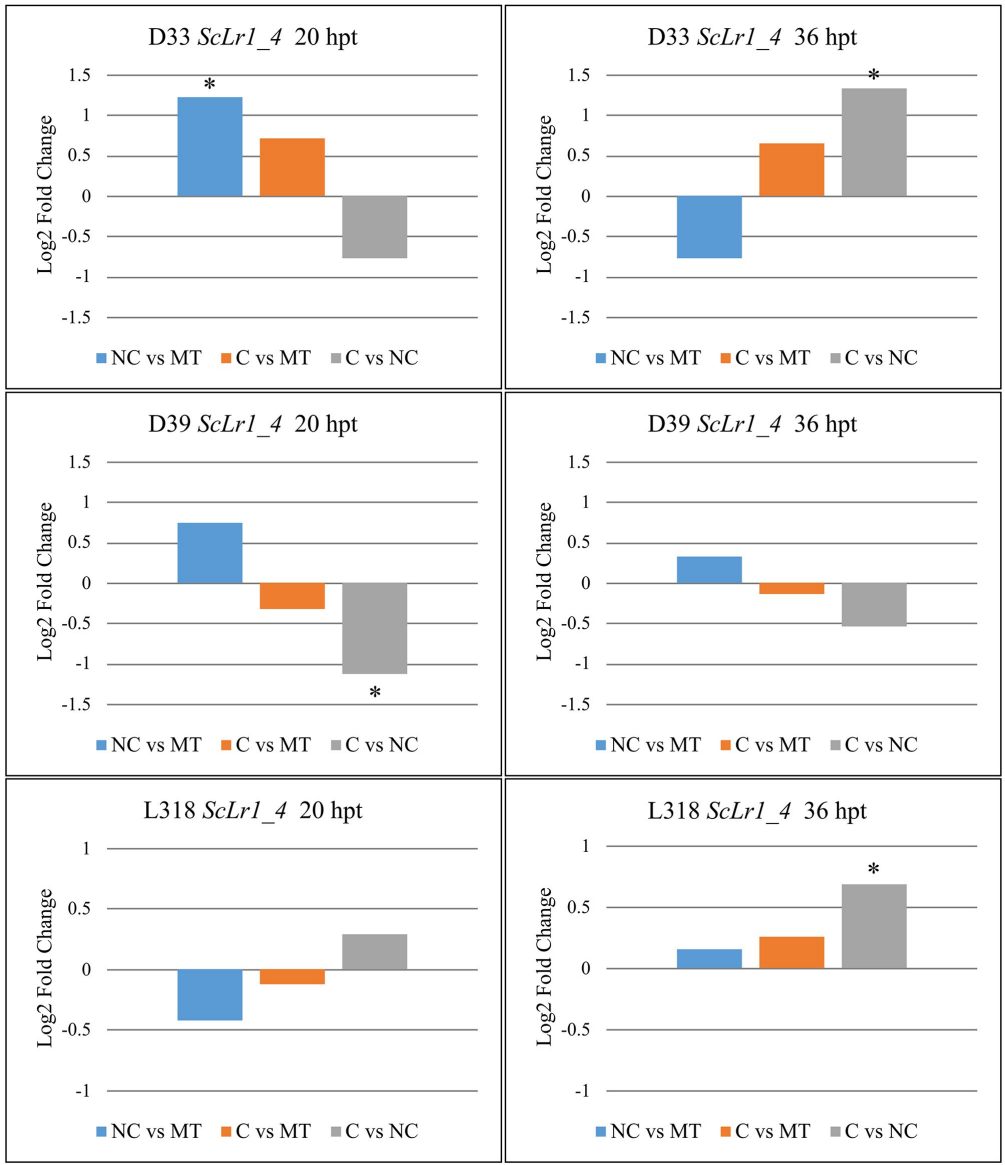
Log2 fold changes in the expression of the *ScLr1_4* gene in rye lines D33, D39, and L318 after treatment with different *Prs* isolates (compatible [C] or non-compatible [NC] strains) relative to the other treatment and a mock-treated control. Plant material for the expression analysis was collected twice: At 20 and 36 hpt. Asterisks indicate significant differences in expression within a given comparison (*p* < 0.05).

In contrast to RT-qPCR results showing that *ScLr1_4* gene expression was significantly upregulated at 20 hpt in D33 treated with a non-compatible *Prs* strain, RNA-seq uncovered no differences in expression levels between mock and either *Prs* treatment, regardless of strain type (Fig 3). In a few cases, however, a significant difference in *ScLr1_4* expression was observed between compatible and non-compatible strain treatments. The gene was upregulated in two comparisons (D33 C vs. NC at 36 hpt, and L318 C vs. NC at 36 hpt) and downregulated in one (D39 C vs. NC at 20 hpt). In line D39 at 36 hpt, *ScLr1_4* expression was reduced after treatment with a compatible strain relative to non-compatible strain, similar to the RNA-seq results, but this difference was not statistically significant.

**Fig 4:**
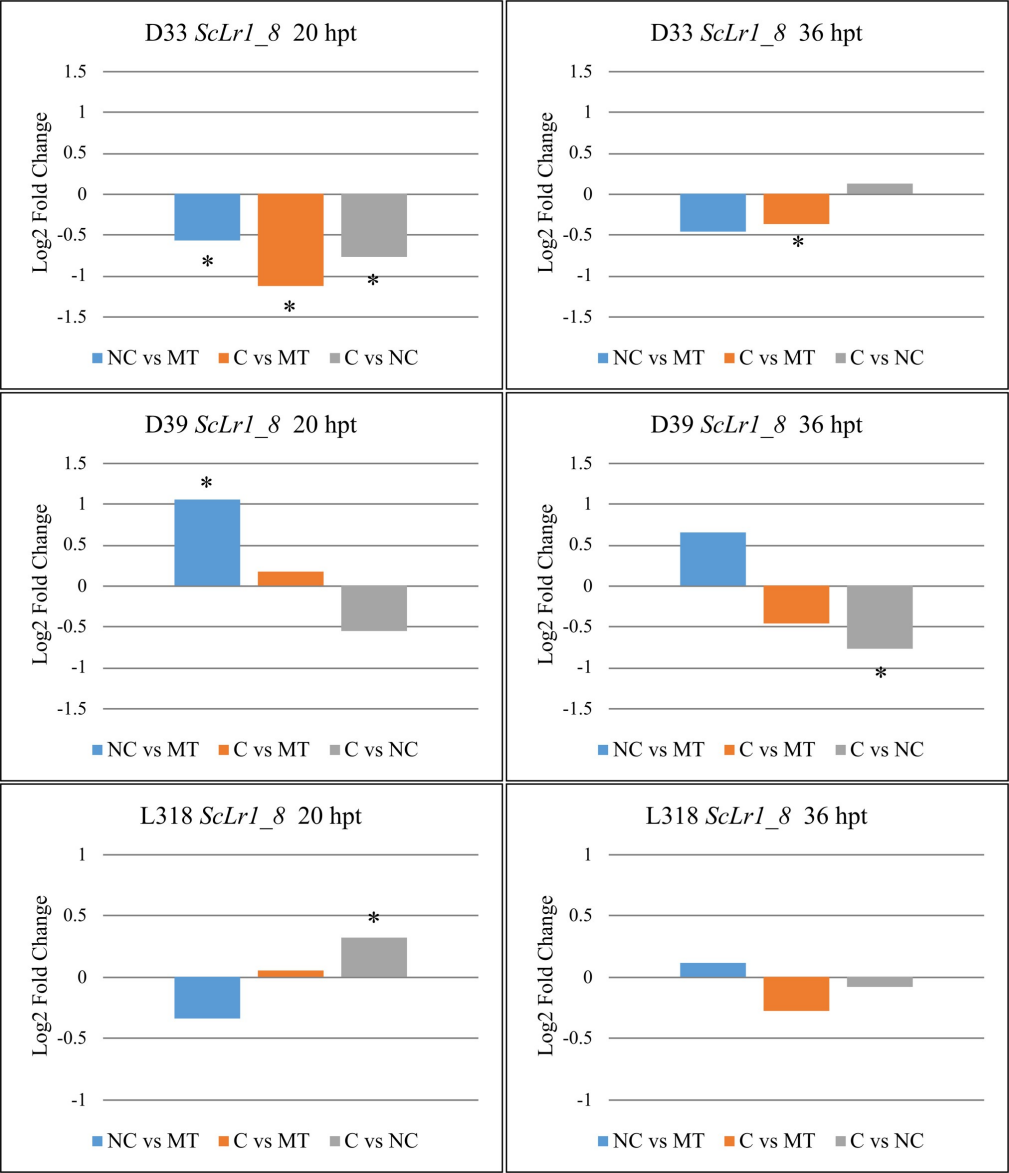
Log2 fold changes in the expression of the *ScLr1_8* gene in rye lines D33, D39, and L318 after treatment with different *Prs* isolates (compatible [C] or non-compatible [NC]) relative to the other treatment and a mock-treated control. Plant material for the expression analysis was collected twice: At 20 and 36 hpt. Asterisks indicate significant differences in expression within a given comparison (*p* < 0.05).

The expression of *ScLr1_8* was downregulated in rye line D33, relative to the mock-treated control, after treatment with either of the *Prs* strains (Fig 4). According to the RNA-seq analysis, however, significant downregulation of this gene was observed only at 20 hpt, whereas RT-qPCR also revealed significant downregulation at 36 hpt, but only in the case of compatible-strain treatment. The opposite was true according to the RNA-seq data for line D39, where treatment with a non-compatible strain upregulated *ScLr1_8* gene expression compared with the control. The RNA-seq analysis also indicated that *Prs* treatment had no significant effect on *ScLr1_8* expression in L318 compared with the mock treatment. Noteworthily, the two *Prs* strains had significantly different effects on *ScLr1_8* gene expression in all tested lines, but only at some time points. In lines D33 and D39, the expression of *ScLr1_8* after treatment with a compatible strain was significantly lower compared with the effect of a non-compatible strain, but only at 20 hpt (D33) and 36 hpt (D39). In line L318, in contrast, *ScLr1_8* expression was significantly higher after treatment with a compatible strain vs. a non-compatible strain, but only at 36 hpt.

**Fig 5:**
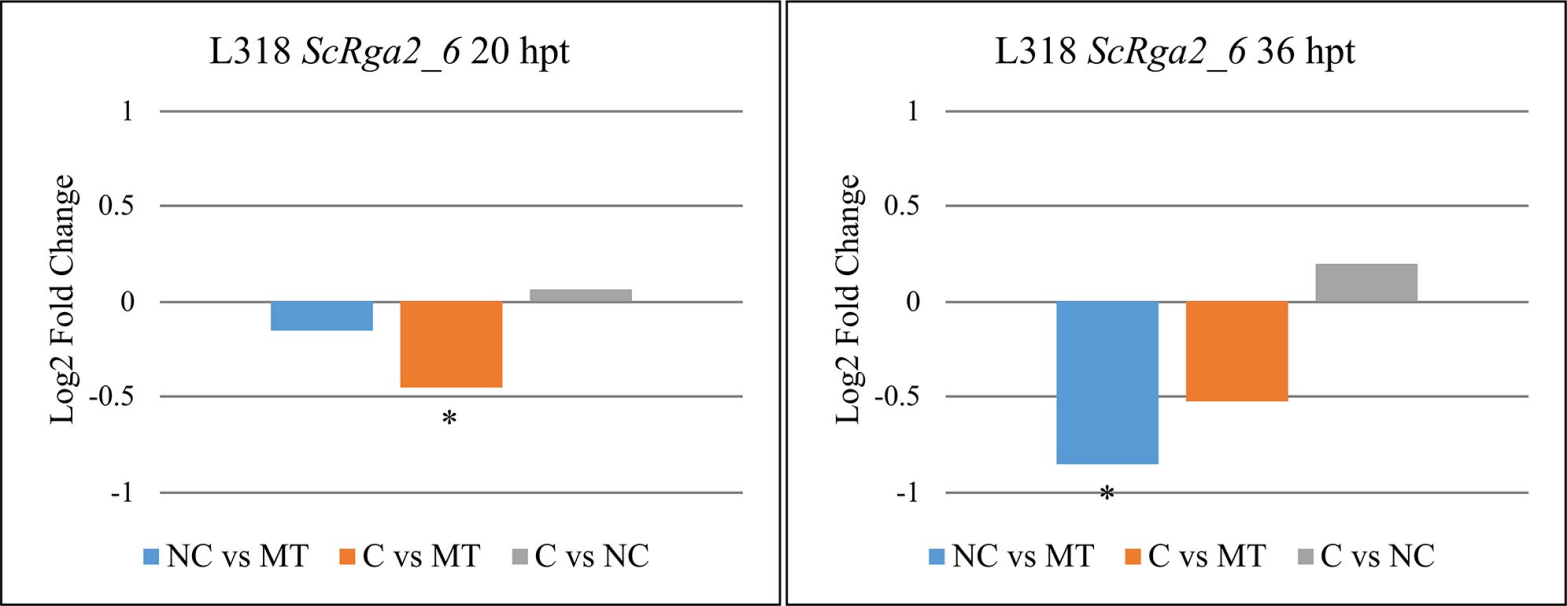
Log2 fold changes in the expression of the *Rga2_6* gene in rye line L318 after treatment with different *Prs* isolates (compatible [C] or non-compatible [NC]) relative to the other treatment and a mock-treated control. The *Rga2_6* gene was absent from rye lines D33 and D39. Plant material for the expression analysis was collected twice: At 20 and 36 hpt. Asterisks indicate significant differences in expression within a given comparison (*p* < 0.05).

The RT-qPCR analysis indicated that *Rga2_6* gene expression was downregulated in rye line L318 after treatment with *Prs*, an observation similar to the overall RNA-seq results (Fig 5). Unlike the findings of the RNA-seq analysis, however, the decreased expression of this gene was significant only for two comparisons (L318 C vs. MT at 36 hpt, and L318 NC vs. MT at 36 hpt). Furthermore, the type of *Prs* strain (compatible vs. non-compatible) had no significant effect on gene expression based on the RT-qPCR analysis, whereas a significant difference was found at 36 hpt according to the RNA-seq data. Similar to the *ScLr1_3* gene, the expression of *Rga2_6* was analyzed by RT-qPCR only in line L318, as this gene was absent from the other two tested rye lines.

## Discussion

Despite the major economic importance of LR and many years of research devoted to the genetic background of rye resistance to this disease, most relevant genes identified thus far have not been molecularly characterized. In this study, we focused on selected R genes, key elements of PTI, that potentially confer LR resistance on rye. All analyzed genes were orthologs of the wheat genes *Lr1*, *Lr10*, *Lr21*, *Lr22a*, and *RGA2/T10rga2-1A*, which all belong to the NBS-LRR or NLR classes and have a proven role in the immune response to LR.

To achieve our study goal, we used three methods: identification of these orthologs in the recently published rye genome [35] followed by their characterization using various tools and algorithms, transcriptome sequencing (RNA-seq), and validation of the RNA-seq data by RT-qPCR analysis. Such a comprehensive approach was used to study the R-type genes associated with LR in rye for the first time.

One of the most popular bioinformatics tools for finding gene orthologs between different species is NCBI BLAST/BLAST+. In published studies, however, various cut-off criteria for the BLAST results have been used to find gene orthologs. The most commonly used parameter is the percentage of sequence identity. Even if a BLAST hit has approximately 100% identity with a query sequence, however, this similarity often only applies to a small portion, sometimes just a tiny fraction, of the alignment. The returned hit is also usually supported by a low *e*-value, but this parameter is highly dependent on gene length and database size and may thus generate false positives. To recognize rye orthologs among BLAST hits in this study, we therefore used generally accepted criteria, including DNA sequence identity ≥ 70% and *e*-value ≤ 10^−6^ [53]; in addition, we used query coverage per HSP ≥ 30% as a cut-off parameter to eliminate BLAST hits with significant but short alignments, which are often restricted to conserved domain regions.

Using the above criteria, we identified 14 *Lr1* gene orthologs in rye. Similar to wheat *Lr1* genes [27], the *ScLr1* gene family has many members located on different chromosomes. *Lr1* and *ScLr1* genes also have similar structures, NB-ARC domain locations, and sizes. Moreover, *ScLr* genes on chromosome 7R have been previously identified by Vendelbo et al. [9] using resistance-associated markers as candidates for rye orthologs of wheat *Lr1* genes. *Lr1* genes possess a signal peptide, as also has been observed in the closest gene orthologs in rye (*ScLr1_1*–*ScLr1_7*, excluding *ScLr1_6*). Taking all of this information into account, one or more *ScLr* genes very likely have the same function as *Lr1* genes; however, as proposed by Vendelbo et al. [9], this idea still needs to be verified.

In contrast to our discovery of multiple *Lr1* genes, we only found one potential *Lr10* ortholog in the Lo7 rye genome. This *ScLr10* gene is structurally different from its wheat ortholog, as the two genes vary significantly in terms of intron size and encoded protein sequence. *ScLr10* is also missing CC and LRR domains and contains only the NB-ARC domain. Moreover, the functional *Lr10* gene in hexaploid wheat is always located on chromosome 1AS [29], whereas *ScLr10* in rye is located on chromosome 6R; no synteny between these chromosomes was observed [54]. Despite these differences, the CDSs of the two genes share approximately 70% DNA sequence similarity. Like *Lr10*, *ScLr10* is not closely related to any other wheat *Lr* genes [28].

To our surprise, we found 15 gene paralogs of *Rga2* in the rye Lo7 genome. In wheat, a closely related species but with a much more complex genome, only two variants of this gene have been detected [28,29]. Similar to wheat *Rga2*, most *ScRga2* genes are longer than the *Lr10* gene and have a partially duplicated NB-ARC domain [55]. All rye *ScRga2* genes (except for *ScRga2_11*) contain a CC domain in the N-terminal, as in the wheat *Rga2* gene. Both the CDS and the genomic sequence of *ScRga2* genes exhibit extensive length variation. The genomic sequence ranges from 1,080 to 13,329 bp, a difference mainly due to the absence or presence of a large intron frequently containing protein domains related to transposable elements.

Of the remaining genes, only two wheat *Lr21* paralogs: the variants *ScLr21_1* and *ScLr21_2* (however, only the second one codes, probably, for LR21 transmembrane protein) and one *Lr22a* paralog were found in the Lo7 genome.

Given that we aimed to determine if, and to what extent, rye orthologs of wheat R genes are induced by infection, we conducted RNA-seq and RT-qPCR analyses. The RNA-seq approach has been widely used to study genes induced by pathogens, including leaf rust [22,56,57] in wheat, which is a close relative of rye. Unfortunately, neither of the aforementioned studies described in detail the genes that we were interested in, i.e., *Lr1*, *Lr10*, *Lr21*, *Lr22a*, and *RGA2/T10rga2-1A*. To date, research on the expression of R genes that confer resistance to LR in wheat is limited to several chromosomal positions and two genes (*Lr1* and *T10rga1/Lr10* [27,28,34]) and none of these studies used the methods used in the present investigation, i.e. RNA-seq and qRT-PCR.

Four of the *ScLr* genes identified in the Lo7 genome in our study (*ScLr1_3*, *ScLr1_4*, *ScLr1_8*, and *ScRga2_6*) were differentially expressed according to the RNA-seq analysis. Among the three studied rye lines, L318 was the only one carrying all four DEGs. This result is not surprising, as a similar observation has been made in wheat, namely, only 16 of 65 tested European wheat accessions in one study possessed the *Lr1* gene [58]. Moreover, *Rga2* (together with *Lr10*) have been found to be mostly absent in the wheat gene pool [28]. The R genes found in the Lo7 genome in our study include orthologs of wheat *Lr10* and *T10rga2-1A* genes, namely, *ScLr10* and *ScRga2*, respectively, which likely correspond to the same H1 haplotype (*Lr10*+/*T10rga2-1A*+) identified in wheat [28]. In the three rye inbred lines analyzed in this study, however, only one variant, *ScRga2_6*, was differentially expressed—in line L318, and no *ScLr10* paralogs were DEGs. Given the present RNA-seq results, we assume that L318 is the only line among the three tested lines that may harbor a functional *ScRga2* gene and that none of these lines have a *ScLr10* gene, or, alternatively, its expression is blocked. Such a haplotype, i.e., *ScLr10-/ScRga2*+, has not yet been identified and may, like the H2 haplotype in wheat, be associated with disease susceptibility. According to our previous field- and phytotron-based assessments [13,14], line L318 has a very low level of LR resistance. The role of *ScLr10* and *ScRga2* genes in the specific immune response of rye to RB infection thus requires further discussion.

According to the RNA-seq analysis and applied criteria, only two variants of *ScLr1* (*ScLr1_3* and *ScLr1_8*) and one variant of *ScRga2* (*ScRga2_6*) were significantly differentially expressed in six comparisons; in all cases, these genes were downregulated, usually at 36 hpt, after infection with either compatible or incompatible *Prs* isolates. In addition, one variant of *ScLr1*, namely, *ScLr1_4*, was overexpressed in plants of line D39 infected with an incompatible *Prs* isolate compared with plants exposed to a compatible strain. This result is inconsistent with most studies of R genes and their roles in the plant immune system. R genes are the main “players” in the second layer (ETI) of the plant immune system, in which proteins encoded by R genes recognize specific pathogen effectors [15]. Therefore, R genes are usually upregulated after pathogen infection, thereby enhancing their potency in pathogen recognition and defense induction [59], even in incompatible relationships [22]. Nevertheless, as stated in the Introduction, R genes may sometimes be downregulated [23–25] or may not be induced at all, especially in incompatible interactions [26].

One possible explanation for the decreased expression of the studied genes after *Prs* treatment is related to the amount of time from inoculation to plant tissue sampling, which was only 20 and 36 hpt. At this early stage, the effectors secreted by a fungus can reduce the level of R-gene expression. In the case of *Prs*, the period between 20 and 36 hpt is characterized by the highest the number of haustorial mother cells (note: haustoria are specialized feeding structures from which pathogens secrete effectors, including proteins recognized by R genes [16,60,61]). Evidence confirming this hypothesis is that relatively less downregulation of the studied genes occurred after infection with incompatible *Prs* isolates; in particular, the compatible strain caused a greater decrease in expression than the incompatible one in two cases involving *Prs* strains of different pathogenicity: *ScLr1_4* in line D39 at 36 hpt, and *ScRga2_6* in line L318 at 36 hpt. Similarly, Li et al. [22] found that the expression level of the gene *TaLr35PR5* in wheat after infection with *Puccinia triticina* was higher in an incompatible interaction than that in a compatible interaction. The same conclusion was made by Bozkurt et al. [62] with regard to *Puccinia striiformis* f. sp. *tritici*. Incompatible strains either do not produce specific effectors or are less effective against R genes. Other causes, such as small RNA-mediated transcript silencing, alternative splicing (AS), and other transcript modifications, cannot be ruled out, but testing these possibilities requires further research. However, it should be noted that AS is a fundamental mechanism of plant stress tolerance. It may be induced both by biotic and abiotic stresses which allow to activate a rapid defense response. This phenomenon has been observed both in dicots and monocots [63,64]. At present, we can neither exclude nor acknowledge AS as a cause of the decreased expression of the studied genes. We examined, by RT-PCR followed by sequencing of the amplified products, the expression of one gene potentially triggering resistance to LR, *ScLr21_1*, and found that in two unrelated rye inbred lines it undergoes AS (S6 Fig); however, we did not look for this phenomenon in any other identified *ScLr* genes.

The variant *ScLr1_8*, identified as a DEG by RNA-seq of line D33, was not amplified from the gDNA template of this line. This unexpected result is possibly due to some difference in the genomic sequence or structure of this gene between lines D33 and Lo7. Similar to some *ScRGA2* gene paralogs, for instance, the *ScLr1_8* gene in line D33 may contain a long intron with incorporated transposons that prevents DNA amplification. Further research is needed to explain this phenomenon. An analysis of *ScLr1_8* will be complicated, however, because this gene is located on an unknown chromosome (ChrUn) containing contigs in the rye reference genome, and its chromosomal location and surrounding sequences are unknown. Moreover, the *ScLr1* gene family contains many genes with very similar nucleotide sequences.

To validate the results of the RNA-seq analysis of the four R genes, we carried out qRT-PCR. This highly sensitive tool for targeted analysis has been broadly used for validation of transcriptomic data, including those produced by the RNA-seq approach [65–69]. We observed a high convergence between RNA-seq- and qRT-PCR-generated results, similar to the findings of many other researchers [66,67,69]. For example, an independent benchmarking study uncovered a high correlation between the results of RNA-seq and qRT-PCR-produced data, and some authors have even questioned the absolute need for such validation. In particular, Coenye [70] has concluded that “RNA-seq methods and data analysis approaches are robust enough to not always require validation by RT-qPCR and/or other approaches, although there are situations where this may be of added value.” An example of this latter situation occurred in a study of wheat *Rf* genes by Tyrka et al. [68], who observed discrepancies between expression levels of several DEGs determined by RNA-seq vs. RT-qPCR that were possibly due to insufficient designed-primer specificity.

As stated above, all DEGs identified by RNA-seq in the present study were positively verified by qRT-PCR, but many more DEGs were uncovered by the latter method than by the former. For the RT-qPCR analysis, we chose to examine gene expression at two time points and analyzed all comparisons, not just those exhibiting differential expression according to RNA-seq. This approach allowed us to identify 14 new cases of significant differential gene expression, namely, six and eight cases of increased and decreased expression, respectively. Among them, five involved comparisons between control plants and plants infected with either compatible or non-compatible *Prs* isolates. Most DEGs in the comparisons C vs. MT and NC vs. MT were downregulated, which supports our hypothesis that pathogen effectors are responsible for the decreased expression of host genes occurring very early after infection.

One of the genes with significant differential expression according to qRT-PCR, but not RNA-seq, was *ScRga2_6*, which was downregulated in line L318 after infection with a compatible *Prs* strain and especially an incompatible strain. Even though *ScRga2_6* expression was downregulated post-infection, this result indicates the important role of this gene in immune response—at least in the specific interaction of plant genotype × *Prs* strain × time point (hpt). A further insight into the involvement of *ScRga2* can be gleaned from the research of Feuillet et al. [28], who assigned an important role to the *Lr10* gene in the expression of *Lr10*-mediated resistance to LR, as well as the claim of Loutre et al. [29] that the *T10rga2/RGA2* gene plays only an auxiliary role in this process. In addition, lowering the expression of *ScRga2* may favor disease development because the *ScRga2/ScLr10* gene pair will not “work” properly in such a case, which may be manifested by the lack of drastic reduction of its expression.

Several factors may be responsible for the differences between RNA-seq and RT-qPCR results described above. First, RT-qPCR is much more specific than RNA-seq, particularly in regard to newly sequenced, incompletely understood genomes, such as the Lo7 rye genome on which our analyses were based. This divergence in sensitivity is especially true of chromosomes labeled Un, which may be mis-mapped. Additionally, in the case of multigene families, like most of our studied genes, they may be enriched for mis-mapped reads. In such cases, verification by RT-qPCR is crucial. We thus do not share the opinion of Coenye [70] that RNA-seq methods do not require RT-qPCR validation; on the contrary, RT-qPCR can be, as in our case, an essential supplement to RNA-seq data.

Second, we did not observe a universal pattern of gene expression across RNA-seq or RT-qPCR analyses—neither for a given rye genotype, nor for a given gene, time point, or isolate pathogenicity. The relationships uncovered were rather specific to a particular set of factors: plant genotype × *Prs* isolate × time after infection. Such dependencies were expected given the applied, unique, experimental design comprising three different, unrelated rye lines and specific *Prs* strains that were compatible or non-compatible in relation to a given genotype.

In conclusion, we can state the following on the basis of our study results: at least some of the analyzed wheat orthologs (namely, *ScLr1_3*, *ScLr1_4*, *ScLr1_8*, and *ScRga2_6*) participate in the response of rye to LR, despite being mainly downregulated. During disease development, Avr proteins are secreted by LR across the haustorial membrane and can suppress multiple plant defense responses, including the hypersensitive response based on R genes [71]. Bi et al. [72] have reported that the effector PNPi from *Puccinia striiformis* f. sp. *tritici*. suppresses the expressions of two plant defense-responsive genes following infection with *Pseudomonas syringae* [72]. The same interaction may be expected for LR effectors and *ScLr* genes after infection with *Prs*.

Among the aforementioned genes, *ScLr1_4* and *ScLr1_8* may play important roles in the early response (20 hpi) of lines D33 and D39, respectively, to LR. These lines are considered to be resistant to LR under field conditions [14] and after treatment with a semi-compatible *Prs* strain in the laboratory [13]. The lines D33 and D39, bred by Danko Plant Breeding, Ltd., belong to the pool of Polish rye breeding materials prevalently used in current breeding programs focused mainly on fertility; these lines can also serve as a source of resistance to LR. It should be noted that, for many years, Polish rye varieties accounted for a large percentage of the cultivars grown in Europe. The function of these genes should be confirmed (for which both genetic transformation and CRISPR/Cas9-mediated gene editing are required), but in the case of rye, an in-vitro-culture recalcitrant species, this represents a major challenge. Nevertheless, in the future, we intend to attempt this using the virus-induced gene silencing technique, which has previously been verified as effective in rye [73].

## Supporting information

S1 Fig LR infection symptoms in D33 rye inbred line infected with leaf rust isolate 83/2/2.2_5x (compatible) and 1/1.6 (non-compatible), 10 dpi. Dankowskie Skand is used as a control.
(DOCX)

S2 Fig Graphical presentation of identified *in silico* transmembrane helices in Lr21 and ScLr21_1 proteins. The graph was created in TMHMM 2.0.
(DOCX)

S3 Fig Graphical presentation of all experiments. (DOCX)

S4 Fig Graphical presentation of subcellular locations of wheat reference Lr1 and rye ScLr1_1, ScLr1_2, ScLr1_4 ScLr1_6, and ScL1_7 proteins predicted using TargetP v2.0 (Organism: Plant). (DOCX)

S5 Fig Phylogenetic tree of rye ScLr and wheat Lr proteins reconstructed by maximum likelihood using the Phylogeny.fr platform [39]. Branch support values (%) were marked in red and only branches with minimum 50% support were shown. The bar at the bottom of the figure indicate the proportion of site changes along each branch.
(DOCX)

S6 Fig Alternative splicing of ScLr21_1 gene in two unrelated rye inbred lines (L310, SE104). DNA Ladder (M) and the samples comes from the same gel image (irrelevant gel lanes between them were intentionally removed). Prior to the experiment, seeds of two rye inbred lines (L310 and SE104) were initially sown in Petri dishes lined with wet tissue paper and left in the dark for 2 days at 22°C. Germinating seeds were then transferred into 12-cm diameter plastic pots (10 seedlings per pot) filled with sterilized peat substrate and maintained for 10 days in a growth chamber at 22°C under a 16-h light/8-h dark photoperiod at an illumination intensity of 60 μmol m-2 s-1. RNA isolation and cDNA synthesis was performed as it was written in Material and Methods section. The RT-PCR was performed in Mastercycler® nexus gradient (Eppendorf). The reaction conditions were as follows: 3 min of denaturation at 95°C followed by 35 amplification cycles (30 s at 95°C, 30 s at 60°C and 60 s at 72°C), then 10 min at 72°C and finally–pause at 15°C. Each reaction was done in 20-μl reaction volume consisting of 10 μl of DreamTaq Green PCR Master Mix 2x (Thermo ScientificTM), 8 μl of cDNA (2.5 ng μl-1), 0.5 μl of each primer and 1 μl of nuclease-free water. Products were electrophoretically separated on 2% agarose gel for 75 min at 115 V.
(DOCX)

S1 Table Benzoxazinoid (BX) contents of inbred rye lines D33, D39, and L318. (DOCX)

S2 Table List of RNA-seq libraries. Each RNA-seq library group consisted of three independent libraries, which were biological replicates.
(DOCX)

S3 Table Detailed description of RNA-seq comparisons used to calculate gene expression fold changes. In each RNA-seq comparison below, fold change was calculated as the ratio of the difference between the expression value of component I over the expression value of component II. Each comparison consisted of three biological replicates. Gene expression was analyzed at two time points (20 hpt and 36 hpt).
(DOCX)

S4 Table Primers used in this study. (DOCX)

S5 Table Detailed structural information on wheat *Lr* and rye *ScLr* genes. (DOCX)

S6 Table Prediction of transmembrane helices (TMHs) in predicted amino acid sequence of *Lr* and *ScLr* genes by TMHMM 2.0. (DOCX)

S7 Table Prediction of subcellular locations of *ScLr* proteins with TargetP v2.0 (Organism: Plant). Annotations are as follows: SP, signal peptide; MT, mitochondrial transit peptide (mTP); CH, chloroplast transit peptide (cTP); TH, thylakoidal lumen composite transit peptide (lTP); OTHER, no targeting peptide.
(DOCX)

S8 Table a. Full RNA-seq results for *Lr* genes orthologs (*ScLr*) in all three rye lines. b. Data statistics summary for RNA-seq libraries based on FASTQC v0.11.8 analysis (before adapter trimming).
(XLSX)
