# Supplementary material for: Identification and characteristics of wheat Lr orthologs in three rye inbred lines
Source: PLoS One. 2023 Jul 13;18(7):e0288520. doi: 10.1371/journal.pone.0288520 (PMC10343146; doi:10.1371/journal.pone.0288520)
Supplement: S1 Fig — Dankowskie Skand is used as a control. (DOCX) [file pone.0288520.s001.docx]

Dankowskie Skand


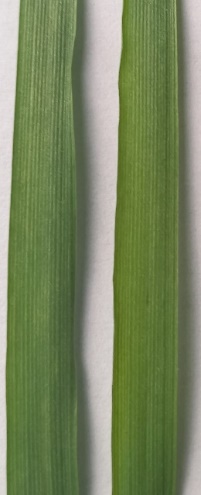

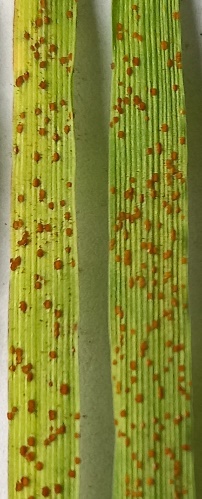


uninfected

83/2/2.2_5x


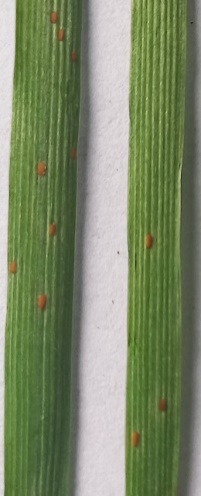

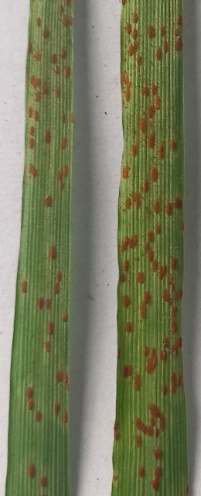


D33

83/2/2.2_5x

1/1.6


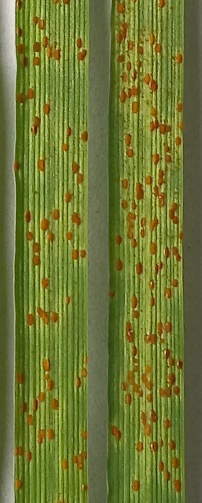


1/1.6

**Figure S1.** **LR infection symptoms in D33 rye inbred line infected with leaf rust isolate 83/2/2.2_5x (compatible) and 1/1.6 (non-compatible), 10 dpi.** Dankowskie Skand is used as a control.
