# Supplementary material for: Identification and characteristics of wheat Lr orthologs in three rye inbred lines
Source: PLoS One. 2023 Jul 13;18(7):e0288520. doi: 10.1371/journal.pone.0288520 (PMC10343146; doi:10.1371/journal.pone.0288520)
Supplement: S2 Fig — The graph was created in TMHMM 2.0. (DOCX) [file pone.0288520.s002.docx]

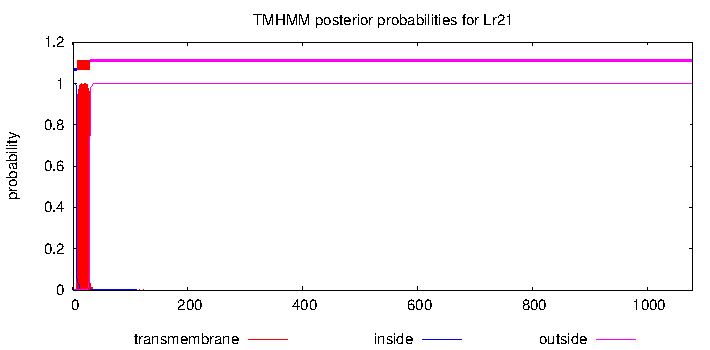


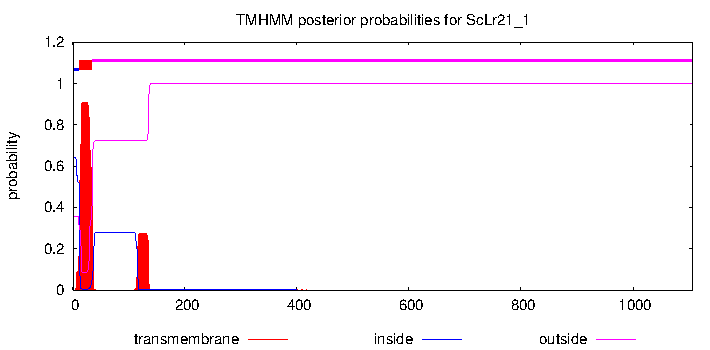


**Figure S2. Graphical presentation of identified *in silico* transmembrane helices in Lr21 and ScLr21_1 proteins.** The graph was created in TMHMM 2.0.
