# Supplementary material for: Identification and characteristics of wheat Lr orthologs in three rye inbred lines
Source: PLoS One. 2023 Jul 13;18(7):e0288520. doi: 10.1371/journal.pone.0288520 (PMC10343146; doi:10.1371/journal.pone.0288520)
Supplement: S3 Fig — (DOCX) [file pone.0288520.s003.docx]

Bioinformatics identification of wheat orthologs of *Lr* genes in rye Lo7 genome

Infected with:

C *Prs*

NC *Prs*

mock treated

3 rye inbred lines:

D33 D39 L318


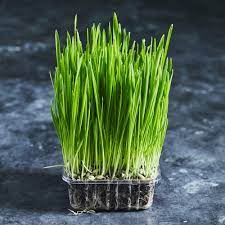

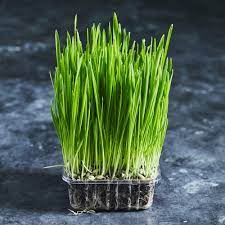

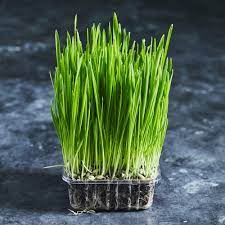

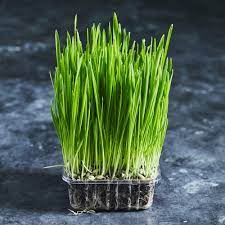

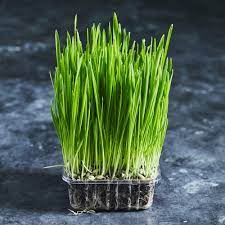

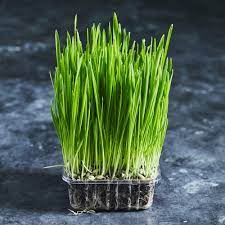

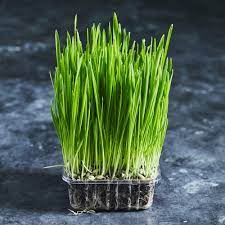

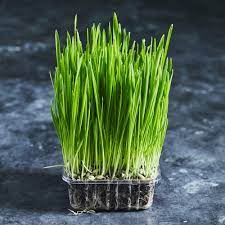


Identifying the wheat orthologs of *Lr1* gene (14 *ScLr1* variants); *Lr10* gene (1 *ScLr10* variant); RGA2/T10rga2-1A gene (15 *ScR~~r~~ga2* variants); *Lr21* gene (2 *ScLr21* variants); *Lr22a* gene (1 *ScLr22a* variant) in rye Lo7 genome and their structural characteristics


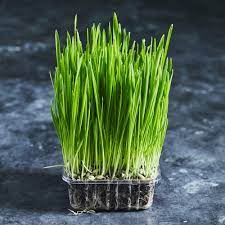


Analysis of the expression of the identified wheat orthologs in 3 rye inbred lines: D33, D39, L318:

- in transcriptomes (RNA-seq data)
- by performing RT-qPCR

Tissue sampled at 20 and 36 hpt and used for:

- RNA-seq
- RT-qPCR

Comparison and discussion of the results obtained

by two types of analyses

**Figure S3. Graphical presentation of all experiments.**
