# Supplementary material for: Identification and characteristics of wheat Lr orthologs in three rye inbred lines
Source: PLoS One. 2023 Jul 13;18(7):e0288520. doi: 10.1371/journal.pone.0288520 (PMC10343146; doi:10.1371/journal.pone.0288520)
Supplement: S4 Fig — (DOCX) [file pone.0288520.s004.docx]

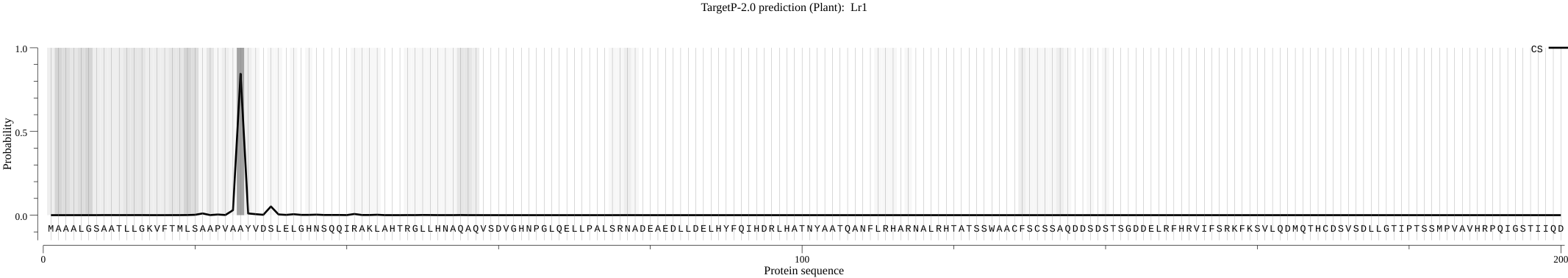


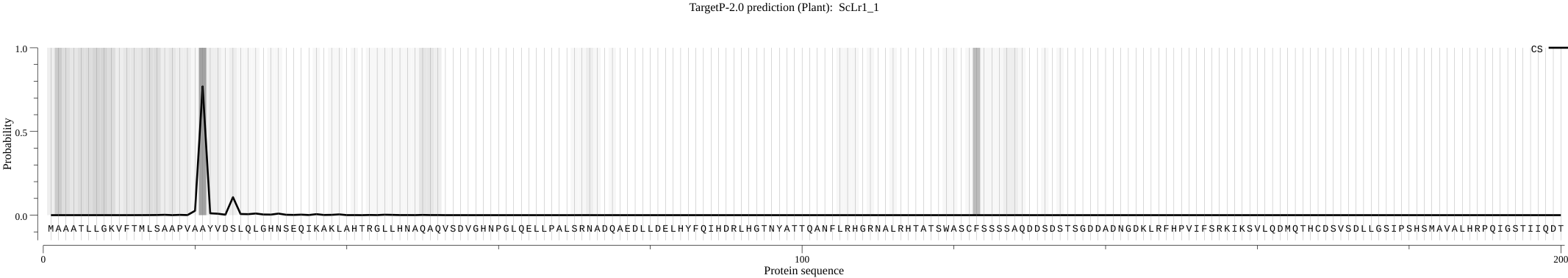


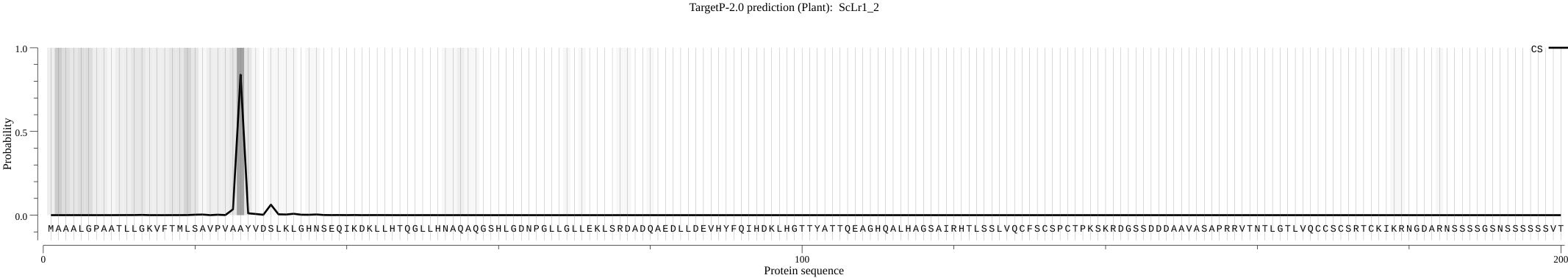


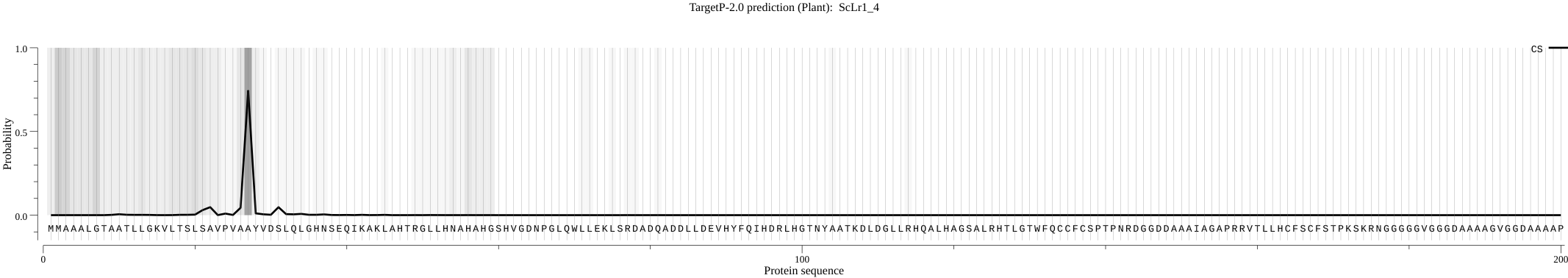


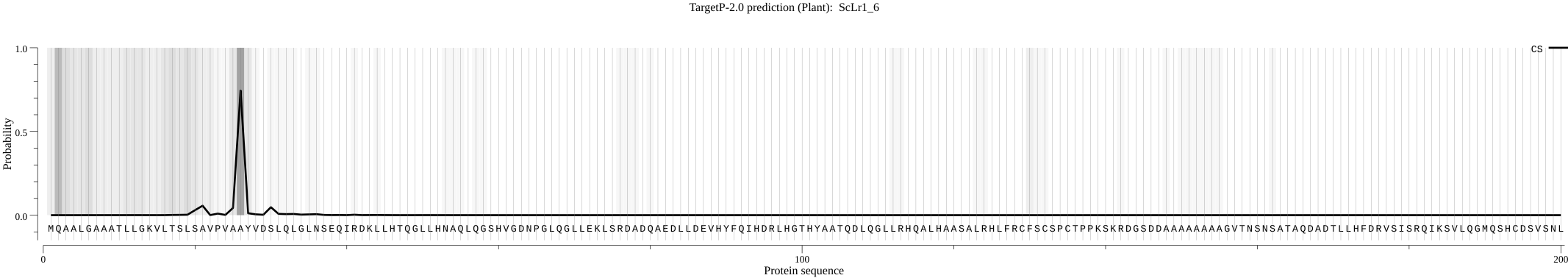


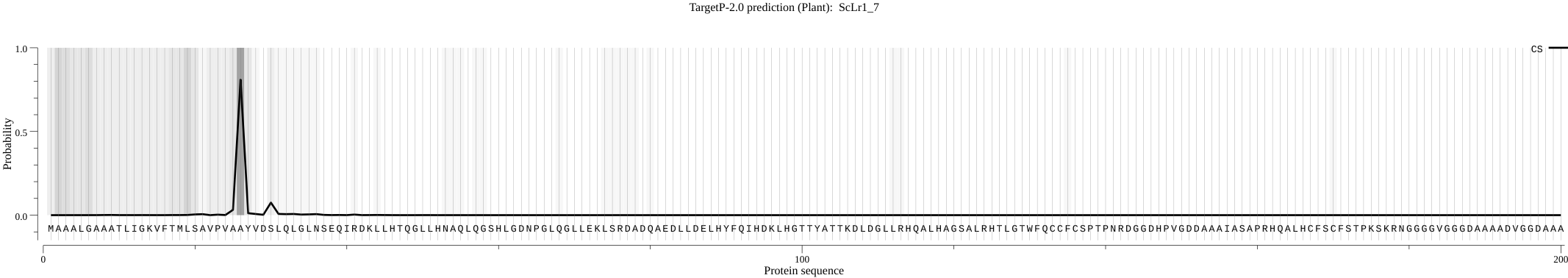


**Figure S4. Graphical presentation of subcellular locations of wheat reference Lr1 and rye ScLr1_1, ScLr1_2, ScLr1_4 ScLr1_6, and ScL1_7 proteins predicted using TargetP v2.0 (Organism: Plant).**
