# Supplementary material for: Identification and characteristics of wheat Lr orthologs in three rye inbred lines
Source: PLoS One. 2023 Jul 13;18(7):e0288520. doi: 10.1371/journal.pone.0288520 (PMC10343146; doi:10.1371/journal.pone.0288520)
Supplement: S5 Fig — Branch support values (%) were marked in red and only branches with minimum 50% support were shown. The bar at the bottom of the figure indicate the proportion of site changes along each branch. (DOCX) [file pone.0288520.s005.docx]

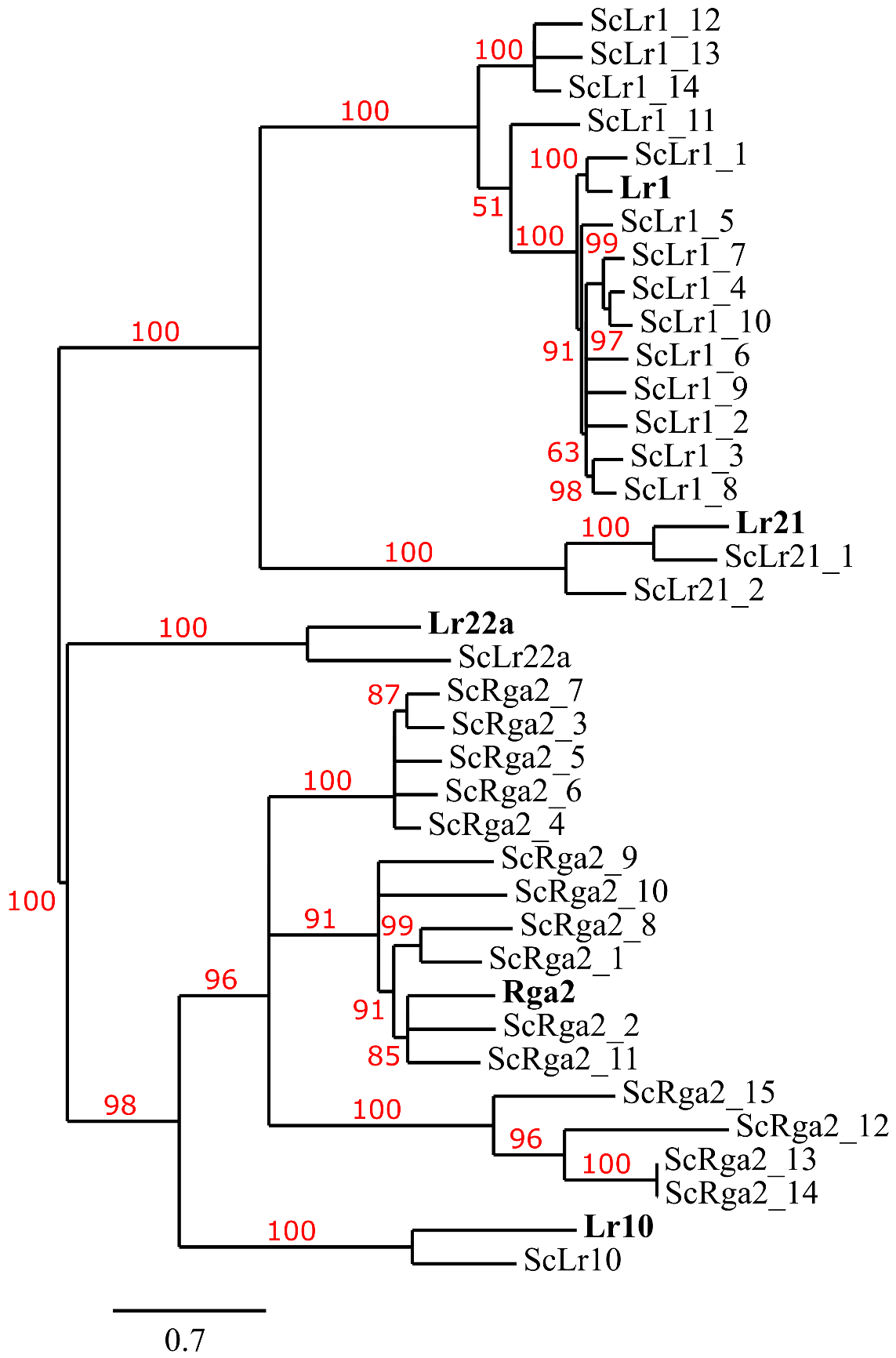


**Figure S5.** **Phylogenetic tree of rye ScLr and wheat Lr proteins reconstructed by maximum likelihood using the Phylogeny.fr platform [39].** Branch support values (%) were marked in red and only branches with minimum 50% support were shown. The bar at the bottom of the figure indicate the proportion of site changes along each branch.
