# Supplementary material for: Identification and characteristics of wheat Lr orthologs in three rye inbred lines
Source: PLoS One. 2023 Jul 13;18(7):e0288520. doi: 10.1371/journal.pone.0288520 (PMC10343146; doi:10.1371/journal.pone.0288520)
Supplement: S6 Fig — DNA Ladder (M) and the samples comes from the same gel image (irrelevant gel lanes between them were intentionally removed). Prior to the experiment, seeds of two rye inbred lines (L310 and SE104) were initially sown in Petri dishes lined with wet tissue paper and left in the dark for 2 days at 22°C. Germinating seeds were then transferred into 12-cm diameter plastic pots (10 seedlings per pot) filled with sterilized peat substrate and maintained for 10 days in a growth chamber at 22°C under a 16-h light/8-h dark photoperiod at an illumination intensity of 60 μmol m-2 s-1. RNA isolation and cDNA synthesis was performed as it was written in Material and Methods section. The RT-PCR was performed in Mastercycler® nexus gradient (Eppendorf). The reaction conditions were as follows: 3 min of denaturation at 95°C followed by 35 amplification cycles (30 s at 95°C, 30 s at 60°C and 60 s at 72°C), then 10 min at 72°C and finally–pause at 15°C. Each reaction was done in 20-μl reaction volume consisting of 10 μl of DreamTaq Green PCR Master Mix 2x (Thermo ScientificTM), 8 μl of cDNA (2.5 ng μl-1), 0.5 μl of each primer and 1 μl of nuclease-free water. Products were electrophoretically separated on 2% agarose gel for 75 min at 115 V. (DOCX) [file pone.0288520.s006.docx]

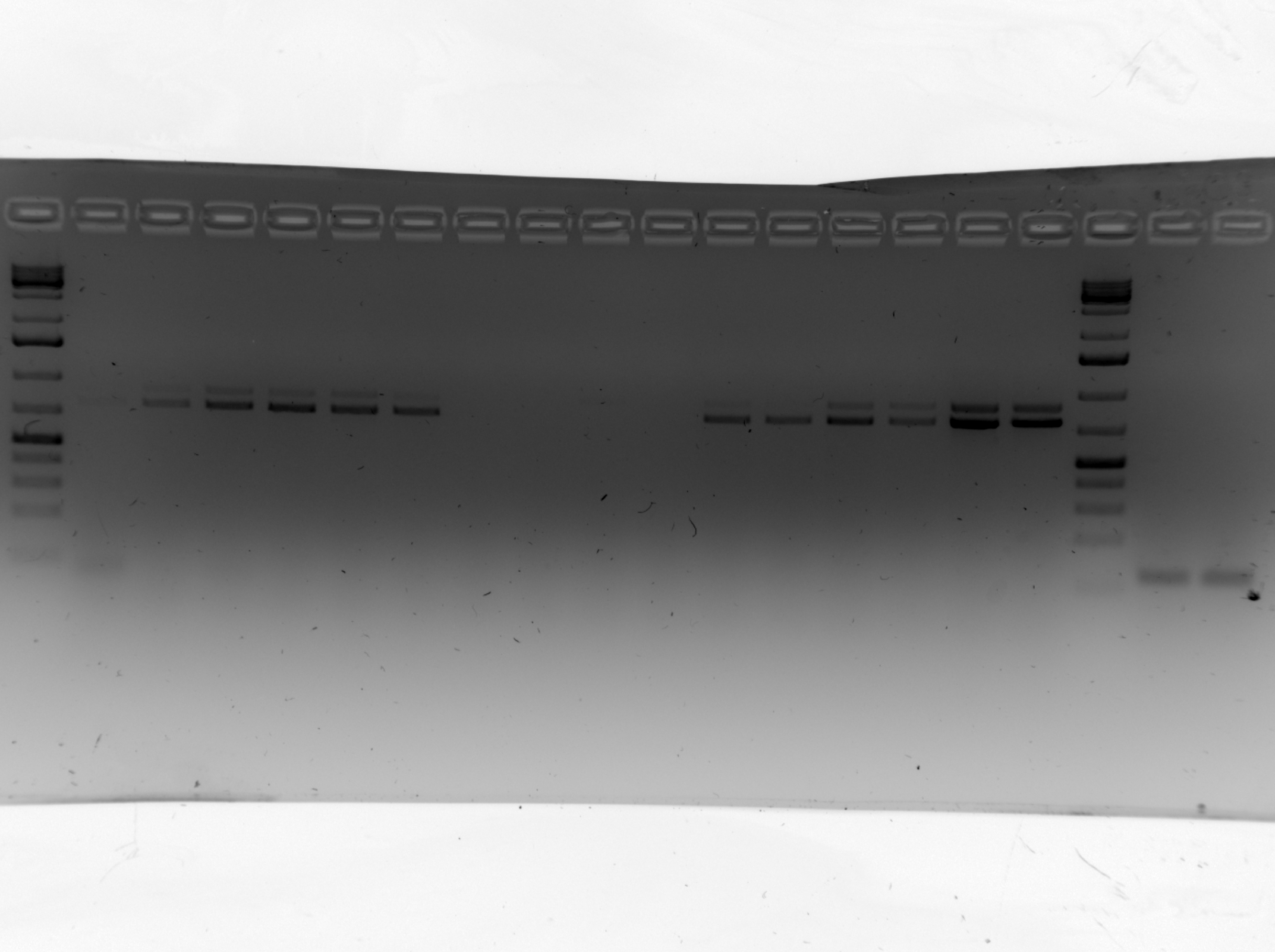

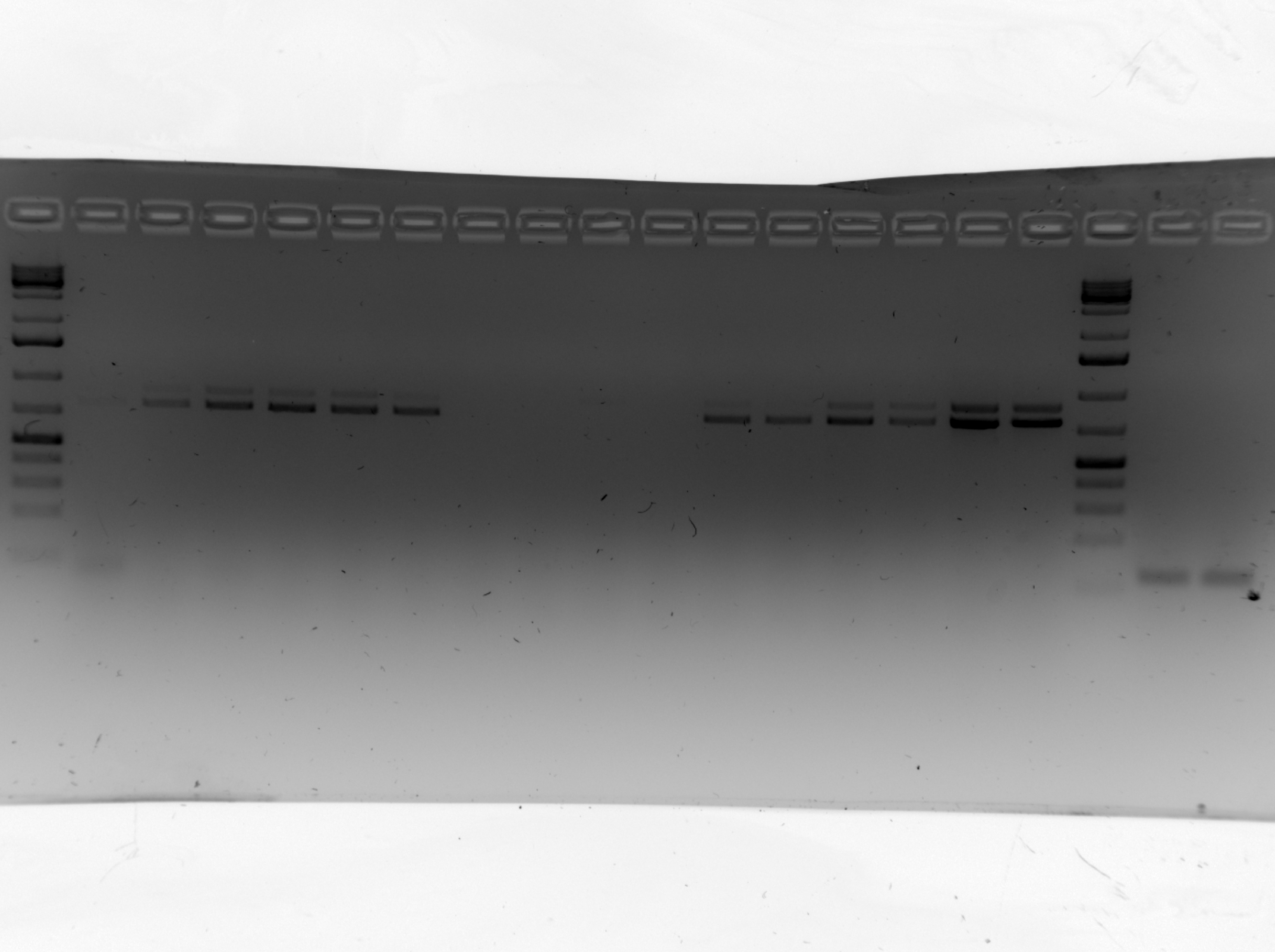


500 bp

700 bp

1000 bp

*ScLr21_1 var2* (intron retention)

*ScLr2_1 var1* (intron spliced out)

L310

SE104

M

*ScLr21_1* DNA

Intron with in-frame STOP codon

RT-PCR product (*Lr21_1 var2*) *–* intron retention

RT-PCR product (*Lr21_1 var1*) *–* intron spliced out

TGA

*ScLr21_1* mRNAs

Exon1

Exon2

NBS domain

| Primer name | Primer sequence |
| --- | --- |
| ScLr21F_13 | CCGGTGACTACCAATGCTGA |
| ScLr21R_13 | AGACATGGATGCACATGATGGT |

**Figure S6. Alternative splicing of *ScLr21_1* gene in two unrelated rye inbred lines (L310, SE104).** DNA Ladder (M) and the samples comes from the same gel image (irrelevant gel lanes between them were intentionally removed). Prior to the experiment, seeds of two rye inbred lines (L310 and SE104) were initially sown in Petri dishes lined with wet tissue paper and left in the dark for 2 days at 22°C. Germinating seeds were then transferred into 12-cm diameter plastic pots (10 seedlings per pot) filled with sterilized peat substrate and maintained for 10 days in a growth chamber at 22°C under a 16-h light/8-h dark photoperiod at an illumination intensity of 60 µmol m^-2^ s^-1^. RNA isolation and cDNA synthesis was performed as it was written in Material and Methods section. The RT-PCR was performed in Mastercycler® nexus gradient (Eppendorf). The reaction conditions were as follows: 3 min of denaturation at 95°C followed by 35 amplification cycles (30 s at 95°C, 30 s at 60°C and 60 s at 72°C), then 10 min at 72°C and finally – pause at 15 ̊C. Each reaction was done in 20-µl reaction volume consisting of 10 μl of DreamTaq Green PCR Master Mix 2x (Thermo ScientificTM), 8 μl of cDNA (2.5 ng μl-1), 0.5 μl of each primer and 1 μl of nuclease-free water. Products were electrophoretically separated on 2% agarose gel for 75 min at 115 V.
