# Supplementary material for: Identification and characteristics of wheat Lr orthologs in three rye inbred lines
Source: PLoS One. 2023 Jul 13;18(7):e0288520. doi: 10.1371/journal.pone.0288520 (PMC10343146; doi:10.1371/journal.pone.0288520)
Supplement: S1 Table — (DOCX) [file pone.0288520.s007.docx]

**Table S1. Benzoxazinoid (BX) contents of inbred rye lines D33, D39, and L318.**

|  | Content^*^ [µg/mg d.m.] of BXs in aerial parts of plants | | | | | | | |
| --- | --- | --- | --- | --- | --- | --- | --- | --- |
| Inbred line | HBOA | GDIBOA | DIBOA | GDIMBOA | | DIMBOA | MBOA | Sum |
| D33 | 0.0153 | 0.1736 | 0.8459 | 0.0019 | 0.0000 | | 0.0015 | 1.0382 |
| D39 | 0.0099 | 0.1755 | 0.4760 | 0.0003 | 0.0000 | | 0.0012 | 0.6639 |
| L318 | 0.0130 | 0.0259 | 1.1788 | 0.0000 | 0.0000 | | 0.0000 | 1.2177 |

^*)^ mean values from two seasons (2014, 2015) and two locations (WULS, Warszawa and West Pomeranian University of Technology Szczecin, Poland).
