# Supplementary material for: Identification and characteristics of wheat Lr orthologs in three rye inbred lines
Source: PLoS One. 2023 Jul 13;18(7):e0288520. doi: 10.1371/journal.pone.0288520 (PMC10343146; doi:10.1371/journal.pone.0288520)
Supplement: S2 Table — Each RNA-seq library group consisted of three independent libraries, which were biological replicates. (DOCX) [file pone.0288520.s008.docx]

**Table S2.** **List of RNA-seq libraries. Each RNA-seq library group consisted of three independent libraries, which were biological replicates.**

| Library  group | Rye line | *Prs* isolate treatment | Collection time (hpt) | No. of libraries | Mean number of RNA-seq pair counts per single library* |
| --- | --- | --- | --- | --- | --- |
| 1 | D33 | Compatible | 20 | 3 | 48498589 |
| 2 | D33 | Compatible | 36 | 3 | 47670872 |
| 3 | D33 | Non-compatible | 20 | 3 | 48741557 |
| 4 | D33 | Non-compatible | 36 | 3 | 48380128 |
| 5 | D33 | Mock-treated control | 20 | 3 | 48367451 |
| 6 | D33 | Mock-treated control | 36 | 3 | 51921189 |
| 7 | D39 | Compatible | 20 | 3 | 51552992 |
| 8 | D39 | Compatible | 36 | 3 | 47224881 |
| 9 | D39 | Non-compatible | 20 | 3 | 46926326 |
| 10 | D39 | Non-compatible | 36 | 3 | 45948063 |
| 11 | D39 | Mock-treated control | 20 | 3 | 49006021 |
| 12 | D39 | Mock-treated control | 36 | 3 | 50858500 |
| 13 | L318 | Compatible | 20 | 3 | 58854326 |
| 14 | L318 | Compatible | 36 | 3 | 46319901 |
| 15 | L318 | Non-compatible | 20 | 3 | 45376969 |
| 16 | L318 | Non-compatible | 36 | 3 | 49397065 |
| 17 | L318 | Mock-treated control | 20 | 3 | 51193799 |
| 18 | L318 | Mock-treated control | 36 | 3 | 43576838 |

*After trimming by Cutadapt v 3.0 (with parameters: -minimum-length = 15 –quality-cutoff = 25).
