# Supplementary material for: Identification and characteristics of wheat Lr orthologs in three rye inbred lines
Source: PLoS One. 2023 Jul 13;18(7):e0288520. doi: 10.1371/journal.pone.0288520 (PMC10343146; doi:10.1371/journal.pone.0288520)
Supplement: S3 Table — In each RNA-seq comparison below, fold change was calculated as the ratio of the difference between the expression value of component I over the expression value of component II. Each comparison consisted of three biological replicates. Gene expression was analyzed at two time points (20 hpt and 36 hpt). (DOCX) [file pone.0288520.s009.docx]

**Table S3. Detailed description of RNA-seq comparisons used to calculate gene expression fold changes.** In each RNA-seq comparison below, fold change was calculated as the ratio of the difference between the expression value of component I over the expression value of component II. Each comparison consisted of three biological replicates. Gene expression was analyzed at two time points (20 hpt and 36 hpt).

| RNA-seq comparison name | Comparison component I | Comparison component II |
| --- | --- | --- |
| D33 C vs MT 20 hpt | line D33 treated with compatible *Prs* (20 hpt) | line D33 mock-treated  (20 hpt) |
| D33 NC vs MT 20 hpt | line D33 treated with non-compatible *Prs* (20 hpt) | line D33 mock-treated  (20 hpt) |
| D33 C vs NC 20 hpt | line D33 treated with compatible *Prs* (20 hpt) | line D33 treated with non-compatible *Prs* (20 hpt) |
| D33 C vs MT 36 hpt | line D33 treated with compatible *Prs* (36 hpt) | line D33 mock-treated  (36 hpt) |
| D33 NC vs MT 36 hpt | line D33 treated with non-compatible *Prs* (36 hpt) | line D33 mock-treated  (36 hpt) |
| D33 C vs NC 36 hpt | line D33 treated with compatible *Prs* (36 hpt) | line D33 treated with non-compatible *Prs* (36 hpt) |
| D39 C vs MT 20 hpt | line D39 treated with compatible *Prs* (20 hpt) | line D39 mock-treated  (20 hpt) |
| D39 NC vs MT 20 hpt | line D39 treated with non-compatible *Prs* (20 hpt) | line D39 mock-treated  (20 hpt) |
| D39 C vs NC 20 hpt | line D39 treated with compatible *Prs* (20 hpt) | line D39 treated with non-compatible *Prs* (20 hpt) |
| D39 C vs MT 36 hpt | line D39 treated with compatible *Prs* (36 hpt) | line D39 mock-treated  (36 hpt) |
| D39 NC vs MT 36 hpt | line D39 treated with non-compatible *Prs* (36 hpt) | line D39 mock-treated  (36 hpt) |
| D39 C vs NC 36 hpt | line D39 treated with compatible *Prs* (36 hpt) | line D39 treated with non-compatible *Prs* (36 hpt) |
| L318 C vs MT 20 hpt | line L318 treated with compatible *Prs* (20 hpt) | line L318 mock-treated (20 hpt) |
| L318 NC vs MT 20 hpt | line L318 treated with non-compatible *Prs* (20 hpt) | line L318 mock-treated (20 hpt) |
| L318 C vs NC 20 hpt | line L318 treated with compatible *Prs* (20 hpt) | line L318 treated with non-compatible *Prs* (20 hpt) |
| L318 C vs MT 36 hpt | line L318 treated with compatible *Prs* (36 hpt) | line L318 mock-treated (36 hpt) |
| L318 NC vs MT 36 hpt | line L318 treated with non-compatible *Prs* (36 hpt) | line L318 mock-treated (36 hpt) |
| L318 C vs NC 36 hpt | line L318 treated with compatible *Prs* (36 hpt) | line L318 treated with non-compatible *Prs* (36 hpt) |
