# Supplementary material for: Identification and characteristics of wheat Lr orthologs in three rye inbred lines
Source: PLoS One. 2023 Jul 13;18(7):e0288520. doi: 10.1371/journal.pone.0288520 (PMC10343146; doi:10.1371/journal.pone.0288520)
Supplement: S4 Table — (DOCX) [file pone.0288520.s010.docx]

**Table S4. Primers used in this study.**

| Primer name | Gene | Primer Sequence | Tm (°C) | Source |
| --- | --- | --- | --- | --- |
| Gene identification | | | | |
| >ScLr1F211 | *ScLr1_3* | CCTCACACCTCCACACTAGC | 58 | This work |
| >ScLr1R211 | *ScLr1_3* | GCAATATCTGAGAGGCCAGC |  |  |
| >qScLr1F225 | *ScLr1_3* | TTTGGCCTACCACAATCTGGA | 58 | This work |
| >qScLr1R225 | *ScLr1_3* | GAGCAAACTGTCGCAAGTTCC |  |  |
| >ScLr1F232 | *ScLr1_4* | GAACTTGGGGCATTGGCTGA | 59 | This work |
| >ScLr1R232 | *ScLr1_4* | TGCTGCTGGAAAGATGAGATACA |  |  |
| >qScLr1F250 | *ScLr1_4* | TGTCGGAGGTATCTTGCACC | 58 | This work |
| >qScLr1R250 | *ScLr1_4* | ACAAGCTTTCTGAATCACATCTTT |  |  |
| >ScLr1F197 | *ScLr1_8* | ACATGTTGAGGAGGATGGACC | 57 | This work |
| >ScLr1R197 | *ScLr1_8* | ACTTCATGCTTGGCTCGTCA |  |  |
| >qScLr1F203 | *ScLr1_8* | ATCTCCGTGTGATGCCCTTG | 56 | This work |
| >qScLr1R203 | *ScLr1_8* | TCCACCTCTTTTGACGACCA |  |  |
| >qScRga2_6_F3 | *ScRga2_6* | AAGTAATAGCGTTGTGGGGCA | 57 | This work |
| >qScRga2_6_R3 | *ScRga2_6* | CGTCGTCGCCATCATCTTCA |  |  |
| Gene identification and sequencing | | | | |
| >ScLr1F212 | *ScLr1_3* | CCCAAGATGTCTCTGCCCTC | 61 | This work |
| >ScLr1R212 | *ScLr1_3* | CAATATCTGAGAGGCCAGCTCTA |  |  |
| >ScLr1F234 | *ScLr1_4* | CAGAACTTGGGGCATTGGCT | 60 | This work |
| >ScLr1R234 | *ScLr1_4* | GATGGTTTGCTGCTGGAAAGATG |  |  |
| >ScLr1F191 | *ScLr1_8* | TGAGCCAAACAGAGCTGGAA | 57 | This work |
| >ScLr1R191 | *ScLr1_8* | CTCAGGGAAGCAGGGAAAGG |  |  |
| >ScRga2_6_F11 | *ScRga2_6* | TACTTTCTGCTTCACCCGCC | 59 | This work |
| >ScRga2_6_R11 | *ScRga2_6* | CTCTTGAAATCACAAGCCAAACC |  |  |
| Gene identification and RT-qPCR | | | | |
| >qScLr1F224 | *ScLr1_3* | GGTTGTTAGTGGGTATGGCG | 57 | This work |
| >qScLr1R224 | *ScLr1_3* | CAGGGAGCAAACTGTCGCAA |  |  |
| >qScLr1F242 | *ScLr1_4* | GGTACCGTTGGTCCTAGCTG | 59 | This work |
| >qScLr1R242 | *ScLr1_4* | ACGCCATCTAGAATGAGTTTCTT |  |  |
| >qScLr1F206 | *ScLr1_8** | TGGTCGTCAAAAGAGGTGGAA | 57 | This work |
| >qScLr1R206 | *ScLr1_8** | CCATCGCATCTTCCGACAGT |  |  |
| >qScLr1F_321 | *ScLr1_8** | CCCTTGTCGGAGGCATGT | 52 | This work |
| >qScLr1R_321 | *ScLr1_8** | GCATCTTCCCACAGCCAT |  |  |
| >qScRga2_6_F2 | *ScRga2_6* | GTAATAGCGTTGTGGGGCAC | 57 | This work |
| >qScRga2_6_R2 | *ScRga2_6* | CGTCGTCGCCATCATCTTCA |  |  |
| RT-qPCR housekeeping genes | | | | |
| >qScActF | RT-qPCR reference | CCCCTTTGAACCCAAAAGCC | 57 | [13] |
| >qScActR | RT-qPCR reference | GAAAGCACGGCCTGAATAGC | 57 | [13] |
| >qScADP-RFaF | RT-qPCR reference | TCTCATGGTTGGTCTCGATG | 55 | [51] |
| >qScADP-RFaR | RT-qPCR reference | GGATGGTGGTGACGATCTCT | 57 | [51] |

* - owing to sequence differences, the following primers were used in RT-qPCR analyses: for rye line L318, primers >qScLr1F206 together with >qScLr1R206; for rye lines D33 and D39, primers >qScLr1F_321 together with >qScLr1R_321.
