# Supplementary material for: Identification and characteristics of wheat Lr orthologs in three rye inbred lines
Source: PLoS One. 2023 Jul 13;18(7):e0288520. doi: 10.1371/journal.pone.0288520 (PMC10343146; doi:10.1371/journal.pone.0288520)
Supplement: S5 Table — (DOCX) [file pone.0288520.s011.docx]

**Table S5. Detailed structural information on wheat *Lr* and rye *ScLr* genes.**

| Gene ID | Gene  name | Chromo-  some | Gene length (bp) | CDS length (bp) | Introns length (bp) | No. of exons | No. of introns |
| --- | --- | --- | --- | --- | --- | --- | --- |
| EF439840 | *Lr1* | 5DL | 4035 | 4035 | 0 | 1 | 0* |
| SECCEUnv1G0527350 | *ScLr1_1* | chrUn | 3852 | 3852 | 0 | 1 | 0 |
| SECCEUnv1G0527300 | *ScLr1_2* | chrUn | 4251 | 4251 | 0 | 1 | 0 |
| SECCE7Rv1G0454480 | *ScLr1_3* | chr7R | 4511 | 4164 | 347 | 2 | 1 |
| SECCE7Rv1G0454510 | *ScLr1_4* | chr7R | 4317 | 4317 | 0 | 1 | 0 |
| SECCE7Rv1G0454380 | *ScLr1_5* | chr7R | 4227 | 4227 | 0 | 1 | 0 |
| SECCE7Rv1G0454390 | *ScLr1_6* | chr7R | 3981 | 3981 | 0 | 1 | 0 |
| SECCE7Rv1G0454490 | *ScLr1_7* | chr7R | 4290 | 4290 | 0 | 1 | 0 |
| SECCEUnv1G0527330 | *ScLr1_8* | chrUn | 4321 | 4215 | 106 | 2 | 1 |
| SECCE7Rv1G0454400 | *ScLr1_9* | chr7R | 3030 | 3030 | 0 | 1 | 0 |
| SECCEUnv1G0527290 | *ScLr1_10* | chrUn | 2667 | 2667 | 0 | 1 | 0 |
| SECCE1Rv1G0053510 | *ScLr1_11* | chr1R | 5156 | 4431 | 725 | 2 | 1 |
| SECCE6Rv1G0447940 | *ScLr1_12* | chr6R | 3627 | 3627 | 0 | 1 | 0 |
| SECCE6Rv1G0447930 | *ScLr1_13* | chr6R | 3618 | 3618 | 0 | 1 | 0 |
| SECCEUnv1G0561580 | *ScLr1_14* | chrUn | 2427 | 2427 | 0 | 1 | 0 |
| AY270157 | *Lr10* | 1AS | 3938 | 2766 | 1172 | 2 | 1 |
| SECCE6Rv1G0440520 | *ScLr10* | chr6R | 1993 | 1938 | 55 | 2 | 1 |
| AY270159 | *Rga2* | 1AS | 4768 | 3510 | 1258 | 3 | 2 |
| SECCE1Rv1G0000300 | *ScRga2_1* | chr1R | 7094 | 3186 | 3908 | 3 | 2 |
| SECCE7Rv1G0483290 | *ScRga2_2* | chr7R | 11721 | 3654 | 8067 | 3 | 2 |
| SECCE6Rv1G0437290 | *ScRga2_3* | chr6R | 12237 | 3588 | 8649 | 3 | 2 |
| SECCE3Rv1G0156830 | *ScRga2_4* | chr3R | 8960 | 3462 | 5498 | 3 | 2 |
| SECCE6Rv1G0437180 | *ScRga2_5* | chr6R | 13329 | 3564 | 9765 | 3 | 2 |
| SECCE6Rv1G0437270 | *ScRga2_6* | chr6R | 9645 | 3588 | 6057 | 3 | 2 |
| SECCE3Rv1G0156840 | *ScRga2_7* | chr3R | 11445 | 3489 | 7956 | 5 | 4 |
| SECCE4Rv1G0265930 | *ScRga2_8* | chr4R | 10268 | 3645 | 6623 | 4 | 3 |
| SECCE4Rv1G0265620 | *ScRga2_9* | chr4R | 5039 | 3825 | 1214 | 3 | 2 |
| SECCE4Rv1G0265660 | *ScRga2_10* | chr4R | 8063 | 3927 | 4136 | 3 | 2 |
| SECCE5Rv1G0307590 | *ScRga2_11* | chr5R | 7662 | 1125 | 6537 | 2 | 1 |
| SECCE1Rv1G0055240 | *ScRga2_12* | chr1R | 3754 | 1797 | 1957 | 3 | 2 |
| SECCE5Rv1G0306370 | *ScRga2_13* | chr5R | 1080 | 1080 | 0 | 1 | 0 |
| SECCE5Rv1G0307570 | *ScRga2_14* | chr5R | 2982 | 1176 | 1806 | 3 | 2 |
| SECCE6Rv1G0437260 | *ScRga2_15* | chr6R | 5505 | 1590 | 3915 | 3 | 2 |
| FJ876280 | *ScLr21* | 1DS | 3690 | 3243 | 447 | 3 | 2 |
| SECCE1Rv1G0002910 | *ScLr21_1* | chr1R | 3456 | 3324 | 132 | 2 | 1 |
| SECCE1Rv1G0002930 | *ScLr21_2* | chr1R | 2148 | 2148 | 0 | 1 | 0 |
| KY064064 | *Lr22a* | 2DS | 2739 | 2739 | 0 | 1 | 0 |
| SECCE4Rv1G0286050 | *ScLr22a* | chr4R | 2574 | 2574 | 0 | 1 | 0 |

* - a 115 bp intron is located in 3’ UTR sequence [27].
