# Supplementary material for: Identification and characteristics of wheat Lr orthologs in three rye inbred lines
Source: PLoS One. 2023 Jul 13;18(7):e0288520. doi: 10.1371/journal.pone.0288520 (PMC10343146; doi:10.1371/journal.pone.0288520)
Supplement: S6 Table — (DOCX) [file pone.0288520.s012.docx]

**Table S6. Prediction of transmembrane helices (TMHs) in predicted amino acid sequence of *Lr* and *ScLr* genes by TMHMM 2.0.**

| Gene name | Protein length (AAs) | Number of predicted TMHs | Expected number of AAs in TMHs | Expected number of AAs in TMHs in the first 60 AAs of the protein | THMs topology |
| --- | --- | --- | --- | --- | --- |
| *Lr1* | 1344 | 0 | 4.04 | 3.89 | o |
| *ScLr1_1* | 1283 | 0 | 0.71 | 0.59 | o |
| *ScLr1_2* | 1416 | 0 | 5.03 | 4.56 | o |
| *ScLr1_3* | 1387 | 0 | 0.18 | 0.07 | o |
| *ScLr1_4* | 1438 | 0 | 2.22 | 2.16 | o |
| *ScLr1_5* | 1408 | 0 | 1.9 | 0.00 | o |
| *ScLr1_6* | 1326 | 0 | 5.32 | 5.24 | o |
| *ScLr1_7* | 1429 | 1 | 15.25 | 14.82 | i5-27o |
| *ScLr1_8* | 1404 | 0 | 0.06 | 0.05 | o |
| *ScLr1_9* | 1009 | 0 | 0.48 | 0.00 | o |
| *ScLr1_10* | 888 | 0 | 0.07 | 0.03 | o |
| *ScLr1_11* | 1476 | 0 | 0.06 | 0.00 | o |
| *ScLr1_12* | 1208 | 0 | 0.46 | 0.06 | o |
| *ScLr1_13* | 1205 | 0 | 0.46 | 0.06 | o |
| *ScLr1_14* | 808 | 0 | 0.47 | 0.00 | o |
| *Lr10* | 921 | 0 | 0.98 | 0.01 | o |
| *ScLr10* | 645 | 0 | 0.01 | 0.00 | o |
| *Rga2* | 1169 | 0 | 0.02 | 0.00 | o |
| *ScRga2_1* | 1061 | 0 | 0.02 | 0.00 | o |
| *ScRga2_2* | 1217 | 0 | 0.06 | 0.00 | o |
| *ScRga2_3* | 1195 | 0 | 0.13 | 0.00 | o |
| *ScRga2_4* | 1153 | 0 | 0.16 | 0.01 | o |
| *ScRga2_5* | 1187 | 0 | 0.01 | 0.01 | o |
| *ScRga2_6* | 1195 | 0 | 0.06 | 0.01 | o |
| *ScRga2_7* | 1162 | 0 | 0.02 | 0.01 | o |
| *ScRga2_8* | 1214 | 0 | 0.01 | 0.00 | o |
| *ScRga2_9* | 1274 | 0 | 0.16 | 0.10 | o |
| *ScRga2_10* | 1308 | 0 | 0.13 | 0.01 | o |
| *ScRga2_11* | 374 | 0 | 0.02 | 0.00 | o |
| *ScRga2_12* | 598 | 0 | 0.01 | 0.00 | o |
| *ScRga2_13* | 359 | 0 | 0.04 | 0.04 | o |
| *ScRga2_14* | 391 | 0 | 0.04 | 0.04 | o |
| *ScRga2_15* | 529 | 0 | 0.02 | 0.01 | o |
| *Lr21* | 1080 | 1 | 22.73 | 22.72 | i7-29o |
| *ScLr21_1* | 1107 | 1 | 25.54 | 19.98 | i12-34o |
| *ScLr21_2* | 715 | 0 | 0.03 | 0.00 | o |
| *Lr22a* | 912 | 0 | 0.01 | 0.01 | o |
| *ScLr22a* | 857 | 0 | 0.28 | 0.28 | o |
