# Supplementary material for: Identification and characteristics of wheat Lr orthologs in three rye inbred lines
Source: PLoS One. 2023 Jul 13;18(7):e0288520. doi: 10.1371/journal.pone.0288520 (PMC10343146; doi:10.1371/journal.pone.0288520)
Supplement: S7 Table — Annotations are as follows: SP, signal peptide; MT, mitochondrial transit peptide (mTP); CH, chloroplast transit peptide (cTP); TH, thylakoidal lumen composite transit peptide (lTP); OTHER, no targeting peptide. (DOCX) [file pone.0288520.s013.docx]

**Table S7. Prediction of subcellular locations of *ScLr* proteins with TargetP v2.0 (Organism: Plant).** Annotations are as follows: SP, signal peptide; MT, mitochondrial transit peptide (mTP); CH, chloroplast transit peptide (cTP); TH, thylakoidal lumen composite transit peptide (lTP); OTHER, no targeting peptide.

| Gene name | Prediction | Other | SP | mTP | cTP | lTP |
| --- | --- | --- | --- | --- | --- | --- |
| *Lr1* | SP | 0.212004 | 0.540167 | 0.040434 | 0.194458 | 0.012937 |
| *ScLr1_1* | SP | 0.377669 | 0.394737 | 0.139171 | 0.081546 | 0.006877 |
| *ScLr1_2* | SP | 0.354477 | 0.634911 | 0.00142 | 0.008257 | 0.000935 |
| *ScLr1_3* | OTHER | 0.976442 | 0.02295 | 0.000173 | 0.000286 | 0.000149 |
| *ScLr1_4* | SP | 0.15622 | 0.542837 | 0.061174 | 0.233872 | 0.005897 |
| *ScLr1_5* | OTHER | 0.998307 | 0.000479 | 0.001198 | 0.000015 | 0.000001 |
| *ScLr1_6* | SP | 0.423195 | 0.531508 | 0.022908 | 0.020978 | 0.001411 |
| *ScLr1_7* | SP | 0.252242 | 0.724777 | 0.006905 | 0.013688 | 0.002388 |
| *ScLr1_8* | OTHER | 0.993751 | 0.005902 | 0.000112 | 0.000138 | 0.000096 |
| *ScLr1_9* | OTHER | 0.99797 | 0.000932 | 0.00108 | 0.000008 | 0.000011 |
| *ScLr1_10* | OTHER | 0.847616 | 0.017045 | 0.0149 | 0.104779 | 0.015659 |
| *ScLr1_11* | OTHER | 0.999559 | 0.000202 | 0.000062 | 0.000074 | 0.000102 |
| *ScLr1_12* | OTHER | 0.995753 | 0.003431 | 0.000338 | 0.000444 | 0.000034 |
| *ScLr1_13* | OTHER | 0.995371 | 0.003825 | 0.000332 | 0.000438 | 0.000034 |
| *ScLr1_14* | OTHER | 0.999966 | 0.000033 | 0.000001 | 0 | 0 |
| *Lr10* | OTHER | 0.9981 | 0.001846 | 0.000046 | 0.000004 | 0.000004 |
| *ScLr10* | OTHER | 0.998238 | 0.000192 | 0.001539 | 0.000001 | 0.000031 |
| *Rga2* | OTHER | 0.998051 | 0.000124 | 0.001806 | 0.000016 | 0.000003 |
| *ScRga2_1* | OTHER | 0.999916 | 0.000055 | 0.000028 | 0.000001 | 0.000001 |
| *ScRga2_2* | OTHER | 0.998097 | 0.000064 | 0.001838 | 0.000001 | 0 |
| *ScRga2_3* | OTHER | 0.99954 | 0.00008 | 0.000349 | 0.000027 | 0.000005 |
| *ScRga2_4* | OTHER | 0.999536 | 0.000253 | 0.000204 | 0.000006 | 0.000002 |
| *ScRga2_5* | OTHER | 0.999622 | 0.000198 | 0.000164 | 0.000012 | 0.000004 |
| *ScRga2_6* | OTHER | 0.999285 | 0.000174 | 0.000462 | 0.000066 | 0.000012 |
| *ScRga2_7* | OTHER | 0.999513 | 0.000334 | 0.00013 | 0.000011 | 0.000012 |
| *ScRga2_8* | OTHER | 0.999924 | 0.000037 | 0.000032 | 0.000004 | 0.000003 |
| *ScRga2_9* | OTHER | 0.999262 | 0.000058 | 0.000542 | 0.000135 | 0.000003 |
| *ScRga2_10* | OTHER | 0.782939 | 0.000341 | 0.212375 | 0.00135 | 0.002995 |
| *ScRga2_11* | OTHER | 0.999962 | 0.000032 | 0.000006 | 0 | 0 |
| *ScRga2_12* | OTHER | 0.999563 | 0.000106 | 0.000276 | 0.00005 | 0.000004 |
| *ScRga2_13* | OTHER | 0.992694 | 0.00228 | 0.004379 | 0.000628 | 0.00002 |
| *ScRga2_14* | OTHER | 0.992694 | 0.00228 | 0.004379 | 0.000628 | 0.00002 |
| *ScRga2_15* | OTHER | 0.999285 | 0.000174 | 0.000462 | 0.000066 | 0.000012 |
| *Lr21* | OTHER | 0.876949 | 0.115311 | 0.002205 | 0.005412 | 0.000122 |
| *ScLr21_1* | OTHER | 0.954893 | 0.044713 | 0.000291 | 0.000055 | 0.000047 |
| *ScLr21_2* | OTHER | 0.999593 | 0.000224 | 0.000173 | 0.000004 | 0.000006 |
| *Lr22a* | OTHER | 0.909195 | 0.000512 | 0.052772 | 0.037455 | 0.000065 |
| *ScLr22a* | OTHER | 0.974412 | 0.004308 | 0.008506 | 0.012668 | 0.000105 |
